# Tuning Fe Spin Moment in Fe–N–C Catalysts to Climb the Activity Volcano via a Local Geometric Distortion Strategy

**DOI:** 10.1002/advs.202203917

**Published:** 2022-09-04

**Authors:** Ruguang Wang, Lifu Zhang, Jieqiong Shan, Yuanyuan Yang, Jyh‐Fu Lee, Tsan‐Yao Chen, Jing Mao, Yang Zhao, Liujing Yang, Zhenpeng Hu, Tao Ling

**Affiliations:** ^1^ Key Laboratory for Advanced Ceramics and Machining Technology of Ministry of Education Tianjin Key Laboratory of Composite and Functional Materials School of Materials Science and Engineering Tianjin University Tianjin 300072 China; ^2^ School of Physics Nankai University Tianjin 300071 China; ^3^ School of Chemical Engineering The University of Adelaide Adelaide SA 5005 Australia; ^4^ National Synchrotron Radiation Research Center Hsinchu 30076 Taiwan; ^5^ Department of Engineering and System Science National Tsing Hua University Hsinchu Taiwan

**Keywords:** Fe–N–C catalysts, oxygen reduction reaction, polar functional groups, spin moment, symmetry breaking

## Abstract

As the most promising alternative to platinum‐based catalysts for cathodic oxygen reduction reaction (ORR) in proton exchange membrane fuel cells, further performance enhancement of Fe–N–C catalysts is highly expected to promote their wide application. In Fe–N–C catalysts, the single Fe atom forms a square‐planar configuration with four adjacent N atoms (*D*_4h_ symmetry). Breaking the *D*_4h_ symmetry of the FeN_4_ active center provides a new route to boost the activity of Fe–N–C catalysts. Herein, for the first time, the deformation of the square‐planar coordination of FeN_4_ moiety achieved by introducing chalcogen oxygen groups (XO_2_, X = S, Se, Te) as polar functional groups in the Fe–N–C catalyst is reported. The theoretical and experimental results demonstrate that breaking the *D*_4h_ symmetry of FeN_4_ results in the rearrangement of Fe 3*d* electrons and increases spin moment of Fe centers. The efficient spin state manipulation optimizes the adsorption energetics of ORR intermediates, thereby significantly promoting the intrinsic ORR activity of Fe–N–C catalysts, among which the SeO_2_ modified catalyst lies around the peak of the ORR volcano plot. This work provides a new strategy to tune the local coordination and thus the electronic structure of single‐atom catalysts.

## Graphical abstract

Herein, a versatile strategy to distort the square‐planar coordination configuration of FeN_4_ by introducing polar functional groups in the carbon matrix of Fe–N–C catalysts is reported. It is believed that this local coordination manipulation strategy will become a general method for adjusting the electronic structure of single‐atom catalysts.

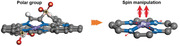

## 1 Introduction

During the past decade, iron–nitrogen–carbon (Fe–N–C) catalysts^[^ ^1^ ^]^ have received intensive attention from academia and industry because of their considerable potential to replace platinum‐based catalysts for cathodic oxygen reduction reaction (ORR) in proton exchange membrane fuel cells.^[^ ^2^, ^3^ ^]^ Significant research efforts have revealed that the active site of Fe–N–C catalyst is FeN_4_ moiety,^[^ ^4^, ^5^, ^6^, ^7^, ^8^, ^9^, ^10^, ^11^ ^]^ which binds with the oxygen intermediates too strongly^[^ ^12^, ^13^, ^14^ ^]^ and down‐shifting the Fe *d*‐orbital energy level can optimize the adsorption energetics.^[^ ^15^, ^16^ ^]^ To achieve this goal, various strategies have been reported, focusing on redistributing the charge of the Fe centers with coordination atoms and carbon matrix.^[^ ^17^, ^18^, ^19^, ^20^, ^21^, ^22^, ^23^ ^]^

Nevertheless, both theoretical calculations and experimental investigations show that even if Fe centers exhibit the same charge state, the ORR catalytic activity of Fe–N–C catalysts varies greatly.^[^ ^6^, ^24^, ^25^, ^26^ ^]^ Similar behavior has also been reported in transition metal oxide catalysts,^[^ ^27^, ^28^, ^29^ ^]^ suggesting that in addition to the charge, the spin of the active metal site (spin state or net spin moment) has a significant effect on catalytic performance. Therefore, spin manipulation^[^ ^30^ ^]^ has been proved as a universal strategy to modulate the electronic structure and optimize the catalytic activity of these catalysts.^[^ ^31^ ^]^ It is recently demonstrated that spin regulation of Fe centers could potentially enhance the ORR performance of Fe–N–C catalyst.^[^ ^32^, ^33^, ^34^, ^35^, ^36^, ^37^ ^]^ However, the physical origin of the spin effect and whether spin state is an appropriate electronic structure descriptor for Fe–N–C catalysts are not clear so far. Moreover, the means of spin regulation remain very limited.

For Fe–N–C catalysts, the central Fe atom forms a square‐planar FeN_4_ coordination with four adjacent N atoms.^[^ ^4^, ^38^ ^]^ Given that structural adjustment is one of the most effective way to modulate the electronic structure of materials,^[^ ^39^, ^40^ ^]^ it is anticipated that distorting the square‐planar coordination of FeN_4_ moiety may alter the spin of the Fe center. Some works have applied this strategy,^[^ ^41^, ^42^ ^]^ either intentionally or not, such as adding axial ligands to the Fe centers,^[^ ^43^, ^44^ ^]^ partially replacing N atoms or reducing the coordination number of N atoms.^[^ ^45^, ^46^ ^]^ However, due to the lack of a guideline in the experimental attempts, the relationship among the distortion of FeN_4_, spin manipulation of the Fe center, and the activity of Fe–N–C catalyst has not been clearly elucidated yet.

Herein, we report a versatile strategy to break the *D*_4h_ symmetry of the FeN_4_ moiety by introducing polar functional XO_2_ groups (X = S, Se, Te) in the carbon matrix of Fe–N–C catalysts. Based on theoretical calculation, synchrotron X‐ray absorption spectroscopy (XAS) and magnetism characterization, we demonstrate that the polar functional XO_2_ groups with varied molecular polarity can effectively regulate the distortion magnitude of FeN_4_ square‐planar coordination. This results in the rearrangement of Fe 3*d* electrons and successful spin moment modulation of the Fe centers, which optimizes the adsorption energetics of ORR intermediates on the Fe–N–C catalyst. As a result, the SeO_2_ group modified Fe–N–C catalyst exhibited an intrinsic activity located at the top of the ORR volcano plots, making it amongst the most active ORR catalysts in acid reported so far.

## 2 Results and Discussion

**Figure 1:**
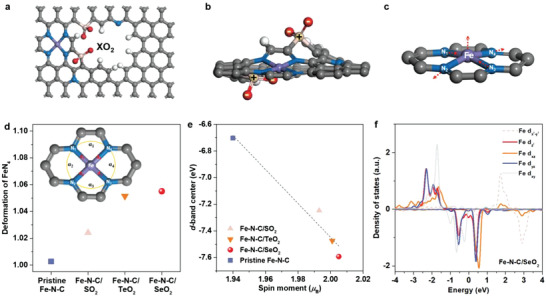
Theoretical investigations on breaking FeN_4_ square‐planar coordination and corresponding changes in electronic structure of Fe centers. a–c) Schematic diagrams of breaking FeN_4_ square‐planar coordination by introducing polar XO_2_ (X = S, Se, and Te) groups. d) Deformation values of FeN_4_ for pristine Fe–N–C and Fe–N–C/XO_2_ catalysts,with the inset showing the definition of the distortion in a FeN_4_ moiety. e) Correlation between *d*‐band center and the spin moment of the Fe centers. f) Projected density of states on Fe *d*‐orbitals in Fe–N–C/SeO_2_.

In this work, to achieve the deformation of FeN_4_ moiety that is embedded into the carbon matrix of Fe–N–C catalyst, polar groups were introduced into the porous carbon to distort the carbon plane through the electrostatic interactions (**Figure** **1**a). Density functional theory (DFT) calculations were firstly performed to evaluate the effect of polar XO_2_ (X = S, Se, and Te) groups on distorting the FeN_4_ square‐planar coordination configuration. It is confirmed that the electrostatic interactions between XO_2_ groups cause a geometric distortion of the FeN_4_ moiety (Figure 1b and Figure S1, Supporting Information). Specifically, the central Fe atom slightly deviates from the planar center, while one diagonal elongated and the other shortened (Figure 1c). Notably, the deformation magnitude of FeN_4_ (*D*_FeN4_) is defined as *D*_*α*_ × *D*_d_, where *D*_*α*_ and *D*_d_ refer to the changes in the N–Fe–N angles (*α*_1_–*α*_4_) and the Fe–N distances (*d*_1_–*d*_4_), respectively (inset of Figure 1d and Figure S2, Supporting Information). For a distorted square‐planar FeN_4_, *D*_*α*_ = 360°/(*α*_1_+*α*_2_+*α*_3_+*α*_4_) > 1 and *D*_d_ = *d*_2_ × *d*_4_/(*d*_1_ × *d*_3_) > 1. As is shown in Figure 1d and Table S1 (Supporting Information), the values of *D*_FeN4_ in the Fe–N–C catalysts investigated follow the order: Fe–N–C/SeO_2_ > Fe–N–C/TeO_2_ > Fe–N–C/SO_2_ > pristine Fe–N–C. In particular, the molecular polarity of the XO_2_ groups is in the order: SeO_2_ > TeO_2_ > SO_2_,^[^ ^47^ ^]^ indicating that the polar XO_2_ groups play a key role in geometric distortion of FeN_4_.

As a result, the electronic structure of Fe center in Fe–N–C catalysts is affected by the distortion of FeN_4_. As shown in Figure 1e, the Fe *d*‐band center of Fe–N–C/XO_2_ moves down relative to that of the pristine Fe–N–C. Remarkably, the change trend of the Fe *d*‐band location is opposite to that of the FeN_4_ deformation magnitude discussed above (Figure 1d). A previous work^[^ ^13^ ^]^ suggested that the change of Fe charge (Δ*Q*_Fe_) may result in the varied location of Fe *d*‐band center. Based on our data (Figures S3 and S4, and Note S1, Supporting Information), Fe–N–C/XO_2_ is not the same case. In‐depth analysis was performed to uncover the underlying reason. We found that the spin moments of the Fe centers in these catalysts were correlated with the Fe *d*‐band center (a linear relationship in Figure 1e), and such correlation originates from the rearrangement of Fe 3*d* electrons. Specifically, when FeN_4_ moiety is distorted by XO_2_ group, some electrons transferred from spin‐down to spin‐up orbitals (Table S2, Supporting Information). This results in an increase in the net spin of the Fe center, which is reasonable that breaking the coordination symmetry of the transition metal will break the degeneracy of its electronic states, leading to electron rearrangement and thus changing its spin moment.^[^ ^31^ ^]^ Moreover, because the energy level of the spin‐down orbitals is higher than that of the spin‐up orbitals (Figure 1f, Figures S5 and S6, Supporting Information), such electron rearrangement will lower the Fe *d*‐band center (Note S2, Supporting Information). Taken together, the introduction of polar XO_2_ groups in Fe–N–C catalysts triggers a geometric distortion of FeN_4_ moiety, leading to an increased Fe spin moment and a lowered Fe *d‐*band center location.

**Figure 2:**
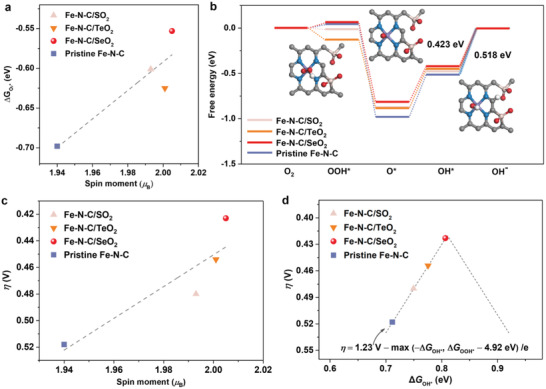
Theoretical predictions of ORR performance. a) Correlation between adsorption free energy of O_2_ (Δ*G*_O2*_) and the Fe spin moment in pristine Fe–N–C and Fe–N–C/XO_2_ catalysts. b) ORR free energy diagram of Fe–N–C/XO_2_ and pristine Fe–N–C catalysts with the inset showing the intermediates OOH*, O*, and OH* adsorbed on Fe–N–C/SeO_2_. c) Correlation between the overpotential (*η*) and the Fe spin moment in Fe–N–C/XO_2_ and pristine Fe–N–C catalysts. d) Volcano plots of *η* versus adsorption energy of OH* (∆*G*_OH*_). The dashed lines were plotted by the equation: *η* = 1.23 V − max (−∆*G*_OH*_, ∆*G*_OOH*_ − 4.92 eV)/*e*,^[^ ^50^, ^51^ ^]^ where ∆*G*_OOH*_ = ∆*G*_OH*_ + 3.30 eV, and ∆*G*_OOH*_ and ∆*G*_OH*_ are the adsorption free energy of OOH* and OH*, respectively.

We further calculated the adsorption free energy of O_2_ (Δ*G*_O2*_) on Fe–N–C catalysts (**Figure** **2**a and Figure S7, Supporting Information). As expected, the O_2_ adsorption on Fe–N–C/XO_2_ is weakened compared with that on pristine Fe–N–C catalyst due to the downshift of Fe *d*‐band center, indicating an optimized ORR activity. Such speculation is verified by the calculated free energy diagrams, with Fe–N–C/SeO_2_ catalyst exhibiting the lowest overpotential (Figure 2b, Figures S8 and S9, Supporting Information). Moreover, the ORR overpotential of Fe–N–C/XO_2_ is correlated with the Fe spin moment. As illustrated in Figure 2c, the adequate linear relationship between the two quantitatively explains the role of Fe spin regulation in enhancing the intrinsic activity of Fe–N–C catalysts. More importantly, it is observed that the intrinsic activity of Fe–N–C/SeO_2_ catalyst lies at the apex of the ORR volcano plot (Figure 2d).

**Figure 3:**
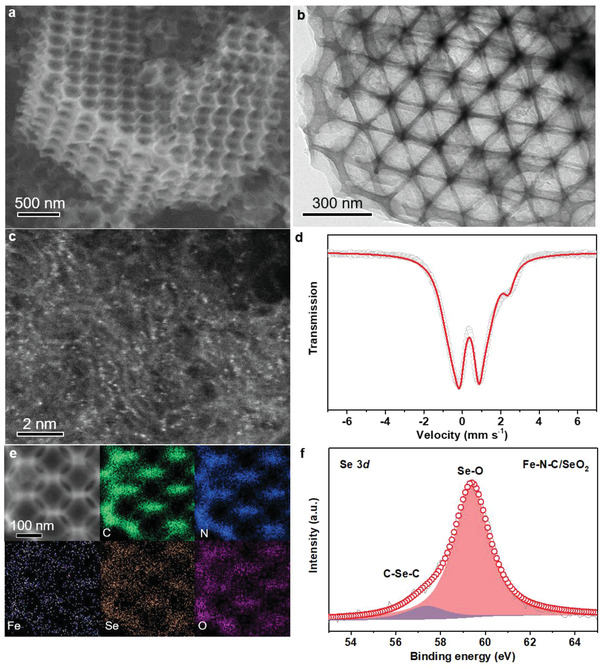
Characterizations of Fe–N–C/XO_2_ catalysts. a) Scanning electron microscopy (SEM) image. b) Transmission electron microscopy (TEM) image. c) HAADF‐STEM image. d) ^57^Fe Mössbauer spectroscopic spectrum. e) EDS elements mappings of C, N, Fe, Se, and O. f) Se 3*d* XPS spectrum. Note that the catalysts characterized here is Fe–N–C/SeO_2_.

Guided by the above theoretical predictions, we fabricated the Fe–N–C/XO_2_ catalysts (Figures S10–S12, Supporting Information). Notably, multiscale pores were specifically constructed in Fe–N–C/XO_2_ catalysts (**Figure** **3**a,b) to ensure that the FeN_4_ active sites are distributed close to the nanopores of the carbon matrix (Figure 3c), making them easy to be distorted by the introduced XO_2_ groups. The formation of single‐atom Fe centers in as‐fabricated Fe–N–C/XO_2_ catalysts was confirmed by X‐ray diffraction (XRD), aberration‐corrected high‐angle annular dark‐field‐scanning transmission electron microscopy (HAADF‐STEM) imaging and Mössbauer spectroscopy (Figure 3c,d, Figures S13–S15 and Table S3, Supporting Information). Energy‐dispersive X‐ray spectroscopic (EDS) elemental mapping shows the homogeneous distribution of C, N, Fe, X (X = S, Se, Te) and O elements in Fe–N–C/XO_2_ catalysts (Figure 3e, Figures S16 and S17, Supporting Information). In addition, the evident signals of oxidized X (XO_2_) in X‐ray photoelectron spectroscopic (XPS) and Fourier transform infrared spectroscopic (FTIR) spectra demonstrate the successful incorporation of polar XO_2_ groups into the carbon matrix of Fe–N–C/XO_2_ catalysts (Figure 3f, Figures S18–S20, and Table S4, Supporting Information).

**Figure 4:**
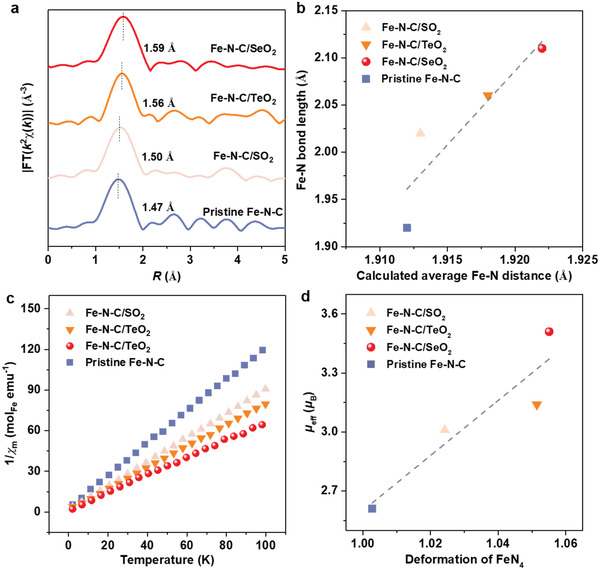
Experimental confirming FeN_4_ distortion and Fe electronic structure change. a) *k* ^2^‐weighted Fourier transform (FT) of Fe *K*‐edge EXAFS spectra of catalysts. b) Correlation between fitted Fe—N bond length by FT‐EXAFS spectra and DFT calculated average Fe–N distance. c) The linear fitting of 1/*χm* (reciprocal of the molar magnetic susceptibility) as a function of temperature performed in the temperature region of 2–100 K. d) Correlation between the effective magnetic moment (*µ*_eff_) of the Fe center and the calculated deformation values of FeN_4_.

The distortion of FeN_4_ *D*_4h_ symmetry by introducing XO_2_ groups was confirmed by Fourier transform (FT) of Fe *K‐*edge extended X‐ray absorption fine structure (EXAFS) spectroscopy (Note S3, Figures S21–S23, Supporting Information). The location of the main peak of the catalysts investigated follows the expected trend: pristine Fe–N–C < Fe–N–C/SO_2_ < Fe–N–C/TeO_2_ < Fe–N–C/SeO_2_ (**Figure** **4**a). This agrees well with the order for the average Fe–N distances obtained by fitting the FT‐EXAFS of these catalysts (Figure 4b, Figures S22, S23, Table S5 and S6, Supporting Information). Importantly, the variation of the average Fe–N distance in Fe–N–C/XO_2_ catalysts correlates well that predicted in our calculation result (Figure 4b).

Since our calculation predicts that the breaking of FeN_4_ *D*_4h_ symmetry would change the spin moments of the Fe centers in Fe–N–C/XO_2_ catalysts (Figure 1e), the magnetic property of the catalysts was experimentally measured using magnetic susceptibility (Figure 4c and Figure S24, Supporting Information). To verify that the observed magnetic change of Fe atoms is due to the FeN_4_ distortion by XO_2_ groups rather than the difference in charge, we kept the Fe oxidation state in these catalysts as the same (Figures S25 and S26, Supporting Information). Moreover, Fe–N–C catalysts were pre‐treated by H_2_/Ar mixture to avoid possible O_2_ adsorption (Figure S27, Supporting Information) and the magnetic measurement was performed under high vacuum conditions. As illustrated in Figure 4d, the measured effective magnetic moment of the Fe centers in these catalysts exhibits the order: pristine Fe–N–C < Fe–N–C/SO_2_ < Fe–N–C/TeO_2_ < Fe–N–C/SeO_2_ (Note S4, Supporting Information), which shows a well correlation with the calculated distortion magnitude of FeN_4_ moiety (Figure 4d). As the geometric and electronic structures of catalysts are essentially correlated, our consistent theoretical and experimental results confirm breaking the *D*_4h_ symmetry of FeN_4_ by introducing polar molecules and successful modulation Fe electronic structure of Fe–N–C catalysts.

**Figure 5:**
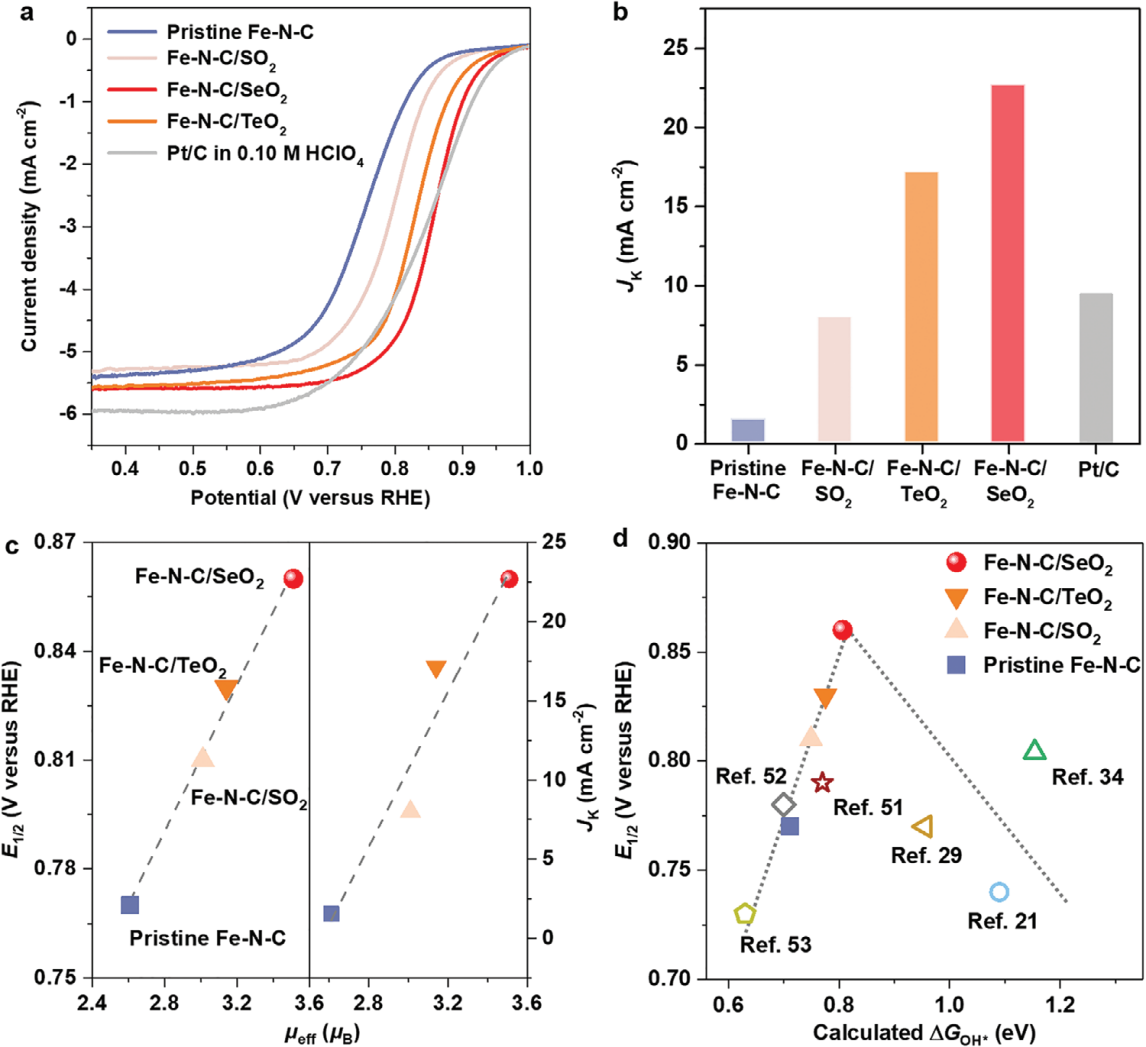
ORR activity of pristine Fe–N–C and Fe–N–C/XO_2_ catalysts. a) Linear scan voltammogram (LSV) curves of Fe–N–C catalysts in O_2_‐saturated 0.50 m H_2_SO_4_ solution and Pt/C catalyst in O_2_‐saturated 0.10 m HClO_4_ at 1600 rpm with a scan rate of 10 mV s^−1^. b) Kinetic current densities (*J*_k_) of catalysts at 0.80 V_RHE_. c) Correlation between *E*_1/2_, *J*_k_, and *µ*_eff_ of the Fe center. d) Correlation between *E*_1/2_ and calculated ∆*G*_OH*_. For comparison, the data points of other non‐precious metal catalysts are plotted according to research reported.^[^ ^21^, ^29^, ^34^, ^51^, ^52^, ^53^ ^]^ The dotted line in (d) is a guide for the eye.

Finally, the catalytic ORR activity of Fe–N–C/XO_2_ catalysts was evaluated. Note that Fe mass loading (≈1.4 wt%), FeN_4_ site density and XO_2_ atomic percentage all exhibited the same values in each catalyst (Figure S28, Tables S4 and S7, Supporting Information). Representative ORR polarization curves of Fe–N–C catalysts in O_2_‐saturated 0.50 m H_2_SO_4_ and 20% Pt/C in O_2_‐saturated 0.10 m HClO_4_ are shown in **Figure** **5**a. Moreover, their kinetic current densities at 0.80 V versus reversible hydrogen electrode (RHE) are summarized in Figure 5b, Figures S29 and S30 (Supporting Information). Obviously, the half‐wave potential (*E*_1/2_) and the kinetic current density (*J*_k_) share the expected trend: pristine Fe–N–C < Fe–N–C/SO_2_ < Fe–N–C/TeO_2_ < Fe–N–C/SeO_2_. Impressively, Fe–N–C/SeO_2_ catalyst features an *E*_1/2_ value of 0.86 V_RHE_ and a *J*_k_ value of 22.7 mA cm^−2^, which are both superior to those of the benchmark Pt/C catalyst. Such excellent performance manifests the Fe–N–C/SeO_2_ catalyst among the most active ORR catalysts in acid reported so far^[^ ^17^, ^48^, ^49^ ^]^ (Table S8, Supporting Information). Moreover, Fe–N–C/SeO_2_ catalyst exhibits a nearly completed conversion from O_2_ to H_2_O with 4e^–^ transfer path together with excellent stability (Figure S31, Supporting Information). Significantly, Fe–N–C/SeO_2_ catalyst also shows excellent ORR activity in 0.10 m KOH (Figure S32, Supporting Information).

Furthermore, we correlate the intrinsic ORR activity of Fe–N–C/XO_2_ catalysts between our experiments and theoretical calculations. The linear relationship observed between the experimental *E*_1/2_ and *J*_k_ (at 0.80 V_RHE_) with the measured Fe spin moment confirms that the net spin moment of the Fe center is an appropriate electronic structure descriptor to evaluate the ORR activity of Fe–N–C catalysts (Figure 5c). Moreover, the experimental *E*_1/2_ is plotted as a function of the computed Δ*G*_OH*_ on Fe–N–C catalysts (Figure 5d). A clear volcano relation with the peak around Δ*G*_OH*_ = 0.81 eV is obtained with Fe–N–C/SeO_2_ catalyst locating around the peak of the volcano plot. The combination of theoretical results and experimental observations demonstrate that we have successfully achieved the optimization of ORR activity in the Fe–N–C catalyst by adjusting the electronic structure of the Fe center, namely its spin moment, which is realized by distorting the coordination configuration of FeN_4_ moiety via introducing XO_2_ groups in the catalyst system.

## 3 Conclusion

In summary, we have theoretically predicted and experimentally verified that breaking the *D*_4h_ symmetry of FeN_4_ moiety by introducing polar functional groups in the Fe–N–C catalysts is a promising route to regulate the electronic structure (spin state) of the Fe center. By this successful Fe electronic structure manipulation, the ORR activity of the Fe–N–C catalysts was greatly enhanced, with Fe–N–C/SeO_2_ locating around the peak of the ORR volcano plot. Moreover, our work establishes the relationship among the deformation magnitude of the FeN_4_ square‐planar coordination, the electronic structure of the Fe center and the activity of Fe–N–C catalyst. We anticipate that the local coordination manipulation strategy achieved by incorporating polar groups will become a general method for adjusting the electronic structure of single‐atom catalysts.

## 4 Experimental Section

### Synthesis of Pristine Fe–N–C and Fe–N–C/XO_2_ Catalysts

Pristine Fe–N–C catalyst was prepared by hard template and ion exchange method. In brief, the pre‐synthesized polystyrene spheres (PSs, 270 nm in diameter) were first assembled into a highly ordered PS template.^[^ ^54^ ^]^ The PS template was then impregnated in precursor solution to obtain ZIF‐8@PS (ZIF: zeolitic imidazolate framework). Afterward, the obtained ZIF‐8@PS was immersed in *N*, *N*‐dimethylformamide (DMF) to decompose the PSs, and heated in a nitrogen atmosphere at 1000 °C for 2 h to obtain the N‐doped carbon matrix. Subsequently, the N‐doped carbon was placed in the center of a quartz tube and anhydrous FeCl_3_ powder was placed 2.5 cm upstream from the tube center. After the quartz tube was outgassed under vacuum, argon (Ar) gas flow (50 sccm) was introduced into the system. The furnace was heated to and kept at 750 °C for 30 min, and then cooled down to room temperature to form the pristine Fe–N–C. Fe–N–C/XO_2_ (X = S, Se, and Te) catalysts were synthesized through high‐temperature processing pristine Fe–N–C in corresponding S, Se, and Te vapor, respectively. Specifically, X powder was placed in the center of a quartz tube, pristine Fe–N–C powder was placed 2.5 cm upstream from the tube center, and then heated in Ar atmosphere to a target temperature (500 °C for S powder, and 600 °C for Se and Te powders), and held for 1 h.

### Materials Characterization

Scanning electron microscope (SEM) and transmission electron microscope (TEM) images were carried out on a Hitachi S‐4800 SEM and a JOEL 2100 TEM, respectively. HAADF‐STEM imaging was performed using a JEOL ARM200F microscope with a STEM aberration corrector operated at 200 kV. The convergent semi angle and collection angle were 21.5 and 200 mrad, respectively. XRD patterns were obtained using a Bruker D8 Advance diffractometer with Cu K*α* radiation operating at 20 kV. Inductively coupled plasma mass spectrometry (ICP‐MS) measurements were carried out on an Agilent Varian 700. ^57^Fe Mössbauer spectroscopy was carried out at room temperature with a proportional counter and a Topologic 500A spectrometer using ^57^Co (Rh) as a *γ*‐ray radioactive source. XAS data, including XANES and EXAFS at Fe *K*‐edge, were collected in total‐fluorescence‐yield mode at ambient air in TLS‐17C beamline at National Synchrotron Radiation Research Center (NSRRC, Hsinchu, Taiwan), in which the electron storage ring was operated at 1.5 GeV with a beam current of 360 mA.

### Magnetic Measurement

Previous works^[^ ^55^, ^56^, ^57^ ^]^ demonstrated that when Fe–N–C catalysts are exposed to air, the adsorption of O_2_ molecules on the Fe center may change the oxidation state of Fe center and cause the distortion of FeN_4_ moiety. In order to avoid such interference, Fe–N–C catalysts investigated were purified by H_2_ and Ar to remove the adsorbed O_2_, confirmed by X‐band electron paramagnetic resonance characterization (Figure S27, Supporting Information). The molar magnetic susceptibility (*χ*_m_) was measured from 2 to 300 K for pristine Fe–N–C and Fe–N–C/XO_2_ with a Superconducting Quantum Interference Device (SQUID) (MPMS XL‐7T, Quantum Design) at a magnetic field of 5000 Oe with a high vacuum of 5 × 10^−5^ torr.

### Fe–N–C Catalysts Purifying

Before XPS and SQUID tests, Fe–N–C catalysts were treated in a tube furnace at 200 °C for 2 h in a H_2_/Ar mixture (10% H_2_). After natural cooling, pure Ar gas was injected into the tube furnace. Under the protection of Ar, the treated samples were immediately placed into sample bags, which were sealed and vacuumed for subsequent XPS and SQUID tests. Note that the sample bags were pre‐cleaned with argon to ensure that they do not contain oxygen and sample transfer during tests was protected with Ar gas.

### Electrochemical Characterization

The electrocatalytic performance of the Fe–N–C catalysts was measured on a WaveDriver 20 electrochemical workstation (Pine Research Instrument), using a three‐electrode system with a graphite rod as the counter electrode, and a saturated calomel as the reference electrode. The catalyst ink was prepared by mixing 3 mg of catalyst and 5 µL of Nafion solution (5 wt%) in 200 µL of deionized water with sonication for 30 min. The prepared ink was dropped onto a glassy‐carbon rotating disk electrode (RDE, 0.196 cm^2^), or a rotating ring disk electrode (RRDE, 0.247 cm^2^) to maintain a catalyst mass loading of 0.8 mg cm^−2^ for all measurements. The LSV curves were recorded at a scan rate of 10 mV s^−1^ until the cyclic voltammetry signals were stable. The H_2_O_2_ yield and electron transfer number were tested on an RRDE.

The accessible Fe–N_4_ site densities of the catalysts investigated were determined by in situ electrochemical method.^[^ ^58^ ^]^ Measurements were performed in a 0.5 m acetate buffer at pH 5.2 for Fe–N–C catalysts using a RDE with the identical catalyst loading mass of 0.27 mg cm^−2^.

### Computational Methods

All the spin‐polarized DFT calculations were conducted with the Vienna Ab‐initio Simulation Package.^[^ ^59^, ^60^, ^61^, ^62^ ^]^ The projector augmented wave pseudopotential^[^ ^63^ ^]^ with the Perdew–Burke–Ernzerhof exchange correlation functional^[^ ^64^ ^]^ was adopted. The plane‐wave kinetic energy cut‐off was set as 400 eV. The energy convergence criterion was set as 10^−5^ eV, and the force was converged to less than 0.03 eV Å^−1^ on each ion. Fe–N–C/XO_2_ catalysts were constructed with the optimized lattice constants (*a* = 19.68 Å, *b* = 12.78 Å, and *c* = 20.00 Å). And *K*‐space was sampled using a 2 × 3 × 1 Monkhorst‐Pack grid. The free energy was computed by the following equation

(1) ΔG=ΔE+ΔZPE−TΔS−eU

where Δ*E* is the energy difference of a given reaction, ΔZPE is the zero‐point energy correction, Δ*S* is the vibrational entropy change at a given temperature *T*, *e* is the elementary charge, and *U* is the electrode potential. The Fe *d*‐band center was calculated by the average energy of electronic *d* states projected onto the Fe atom as following:

(2) $$\varepsilon_{d}=\frac{\int_{-\infty }^{E_{F}}E\rho \left(E\right)dE}{\int_{-\infty }^{E_{F}}\rho \left(E\right)dE}$$

where *E*_F_ is the Fermi energy, *E* is the energy, and *ρ*(*E*) is the density of states of the Fe atom at energy *E*. In recent theoretical works,^[^ ^65^, ^66^, ^67^, ^68^ ^]^ it has been more frequently used since it is a good descriptor within the theoretical framework of the *d*‐band model of surface chemisorptions.

## Conflict of Interest

The authors declare no conflict of interest.

## Supporting information

Supporting Information

## Data Availability

The data that support the findings of this study are available in the supplementary material of this article.
