# Supplementary material for: Tuning Fe Spin Moment in Fe–N–C Catalysts to Climb the Activity Volcano via a Local Geometric Distortion Strategy
Source: Adv Sci (Weinh). 2022 Sep 4;9(31):2203917. doi: 10.1002/advs.202203917 (PMC9631079; doi:10.1002/advs.202203917)
Supplement: Supplementary file 1 — Supporting Information [file ADVS-9-2203917-s001.pdf]

Supplementary Note

Supplementary Note 1. Evaluating the electron-withdrawing effect of XO_2 incorporation on ORR activity.

According to previous works,[1, 2] electron-withdrawing from Fe center is favorable for enhancing the ORR activity. The Bader charge analysis **(Figure S3)** reveals that Fe-N-C/SeO_2 affords the best performance among the three Fe-N-C/XO_2 catalysts investigated, however the Fe center in Fe-N-C/SeO_2 exhibits the richest electrons. Besides, we compared the effects of S and SO_2 doping on the charge, spin state and ORR activity of Fe-N-C catalysts. As shown in **Figure S4**, the charge changes in both the Fe center and the FeN_4 moiety caused by S and SO_2 doping are almost the same, however the Fe spin moment and overpotential of S and SO_2 doped Fe-N-C catalysts vary greatly. Based on these collective results, it is concluded that charge is not the main factor determining the performance of Fe-N-C catalysts in our case.

Supplementary Note 2. Correlation between *d*-band center and spin moment of Fe centers in Fe-N-C catalysts.

As shown in **Figure 1f** and **Figure S5**, the energy level of electron occupied Fe spin-down orbitals (E_{down}) is higher than that of spin-up orbitals (E_{up}) (**Figure S6**). For a FeN_4 moiety without distortion, we assume that there are N_{up} spin up electrons and N_{down} spin down electrons. In this case, the *d*-band center (D_1) can be calculated as,

$$D_1 = \frac{N_{\mathrm{up}}E_{\mathrm{up}} + N_{\mathrm{down}}E_{\mathrm{down}}}{N_{\mathrm{up}} + N_{\mathrm{down}}} \qquad (1)$$

As shown in **Table S2**, as the FeN_4 site is distorted, there are electrons transferred from the higher energy level spin-down orbitals to the lower energy spin-up orbitals. This results in an increasing of the net spin of the system. If the number of transferred electrons is δ, the Fe *d*-band center (D_2) can be obtained by,

$$D_2 = \frac{(N_{\mathrm{up}}+\delta)E_{\mathrm{up}} + (N_{\mathrm{down}}-\delta)E_{\mathrm{down}}}{N_{\mathrm{up}} + N_{\mathrm{down}}} = D_1 + \frac{\delta(E_{\mathrm{up}} - E_{\mathrm{down}})}{N_{\mathrm{up}} + N_{\mathrm{down}}} \qquad (2)$$

Because E_{up} is smaller than E_{down}, D_2 is smaller than D_1. This is just that Fe *d*-band center moves down with an increasing of the Fe spin moment **(Figure 1e)**.

Supplementary Note 3. Verify rationality of evaluating distortion magnitude of FeN_4 by FT-EXAFS.

In this work, all the Fe-N-C catalysts for XAS measurements was pre-treated by Ar/H_2 mixture. As shown in **Figure S26**, the measured Fe *K*-edge XANES spectra of Fe-N-C/XO_2 catalysts is very close to phthalocyanine iron (Fe^{2+}), indicating that the Fe centers in the Fe-N-C catalysts

investigated are dominantly Fe^{2+} without O_2 adsorption. This is in line with our X-band EPR spectra and XPS results **(Figures S25 and S27)**. According to the literature, [3-5] O_2 molecules are possibly adsorbed on some Fe centers of Fe-N-C catalysts during XAS measurements. Our calcualtion results reveal that O_2 adsorption hardly affects the distortaion magnitude of FeN_4 moiety **(Figure S21)**, and our fitting results of FT-EXAFS show that the Fe-N-C/XO_2 catalysts exhibit no significant change in the average Fe-N distance considering O_2 adsorption **(Table S6)**. It can be explained that the calculated O_2 adsorption free energy on Fe-N-C catalysts is about −0.6 eV (**Figure 2a**), which is quite small compared with the ultrahigh bond energy of FeN_4 (−7 ~ −8 eV)[6, 7] in carbon matrix. Therefore, based on the above detailed analysis, the distortion magnitude of FeN_4 in Fe-N-C/XO_2 catalysts can be safely eveluated by FT-EXAFS.

Supplementary Note 4. Analysis of Fe magnetic moments in Fe-N-C catalysts.

The average magnetic moment of Fe-N-C catalysts was measured by using the magnetic molar susceptibility as a function of temperature.[8] Based on the Curie−Weiss law, the experimental data can be fitted using the following equation,

$$\chi_m^{-1} = (T - T_{\mathrm{C}})/C_m \qquad (3)$$

where χ_m is the molar magnetic susceptibility of Fe, C_m is the Curie constant, and T_{C} is the Curie temperature. Based on the obtained value of C_m, the average effective magnetic moment (μ_{eff}) of each Fe atom can be calculated by

$$\mu_{\mathrm{eff}} = 2.82 \cdot C_m^{1/2} \qquad (4)$$

Supplementary Figures

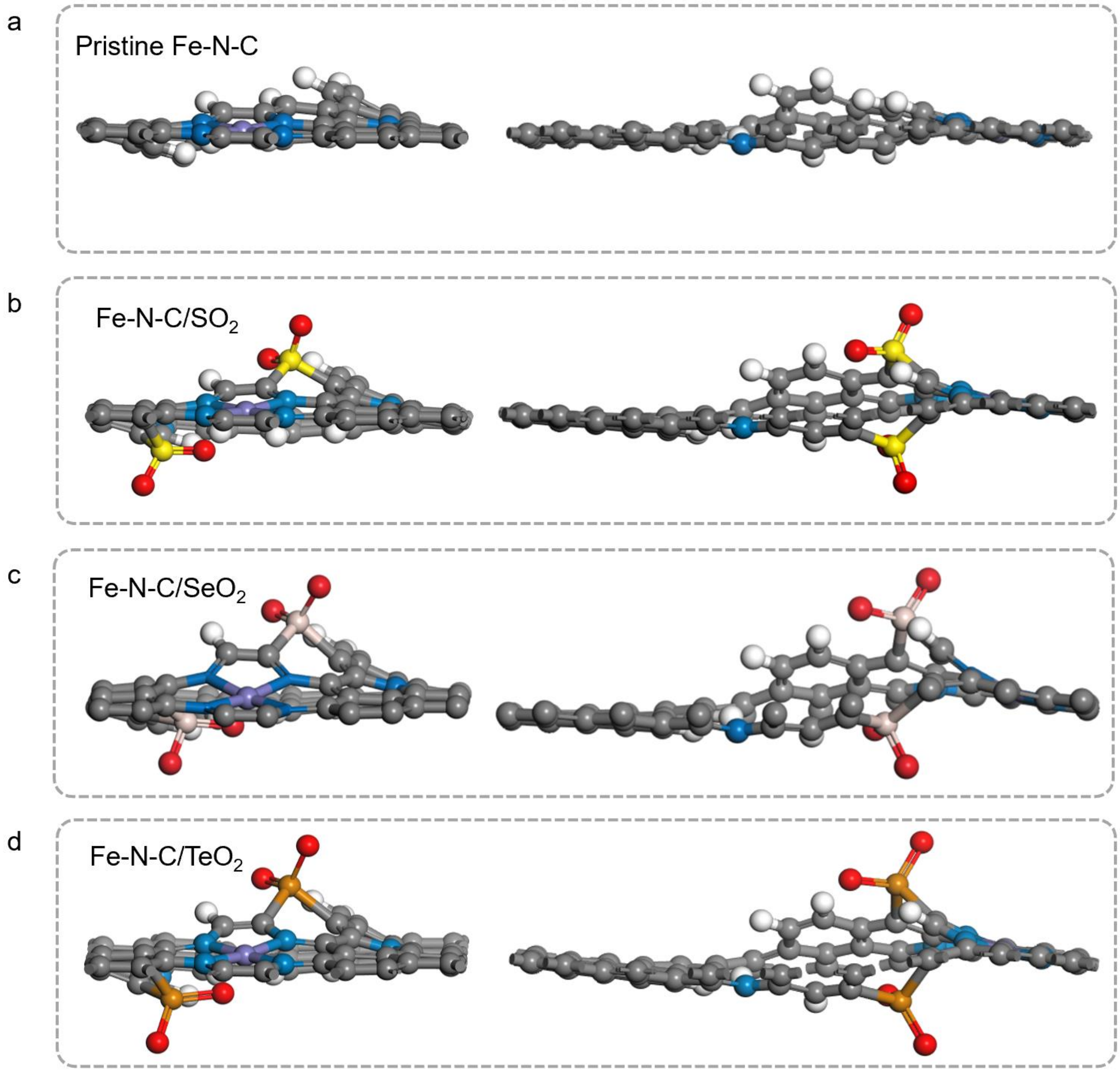

Figure S1. Computationally-optimized geometric structures of (a) pristine Fe-N-C, (b) Fe-N-C/SO_2, (c) Fe-N-C/SeO_2 and (d) Fe-N-C/TeO_2. The grey, red, blue, lilac, yellow, pink, and brown colored balls represent, C, O, N, Fe, S, Se and Te atoms, respectively.

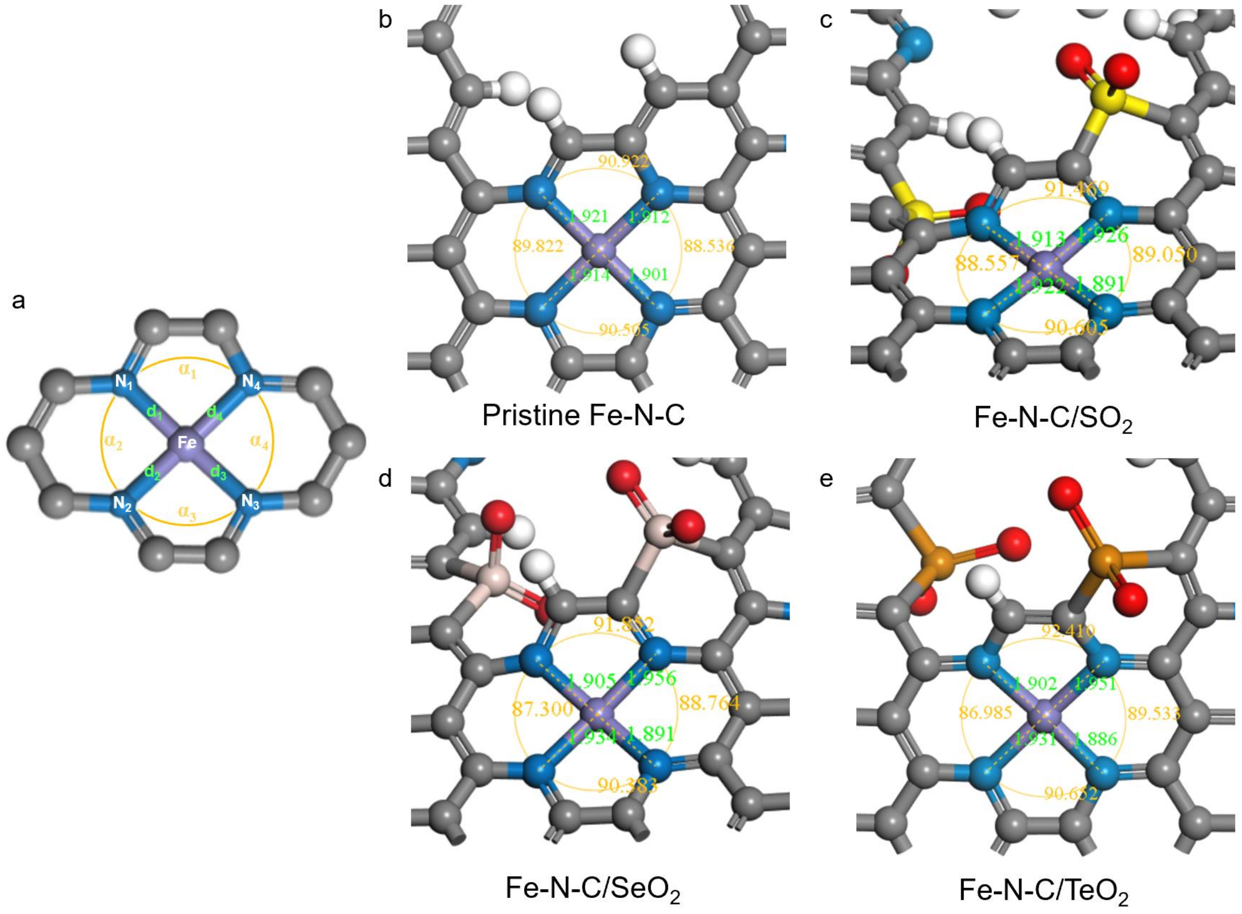

Figure S2. (a) The definition of the distortion of a FeN_4 moiety. (b-e) Calculated deformation of the FeN_4 moiety in pristine Fe-N-C, Fe-N-C/SO_2, Fe-N-C/SeO_2, and Fe-N-C/TeO_2, respectively. Color codes are the same as in **Figure S1**.

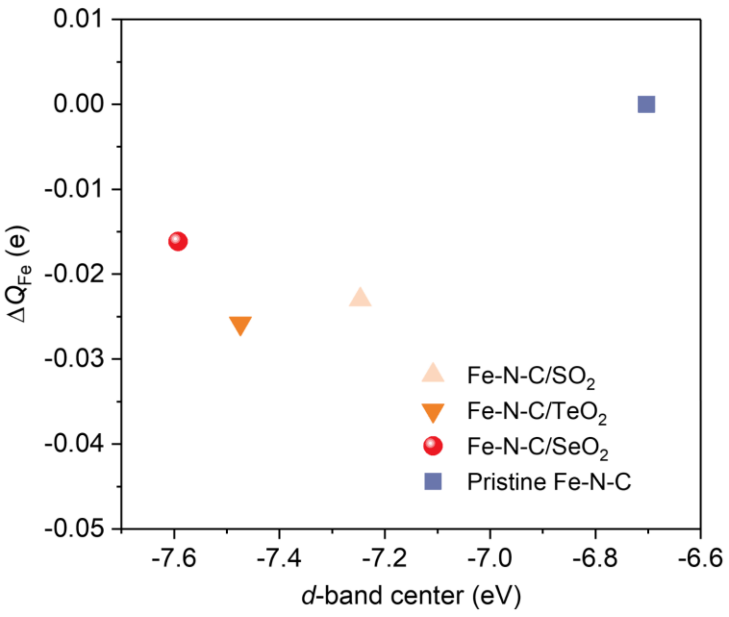

Figure S3. Relationship between the charge change of Fe (ΔQ_{Fe}) and the Fe *d*-band center location for pristine Fe-N-C and Fe-N-C/XO_2 catalysts. $\Delta Q_{Fe} = Q_{Fe}^{\text{Fe-N-C/XO2}} - Q_{Fe}^{\text{pristine Fe-N-C}}$.

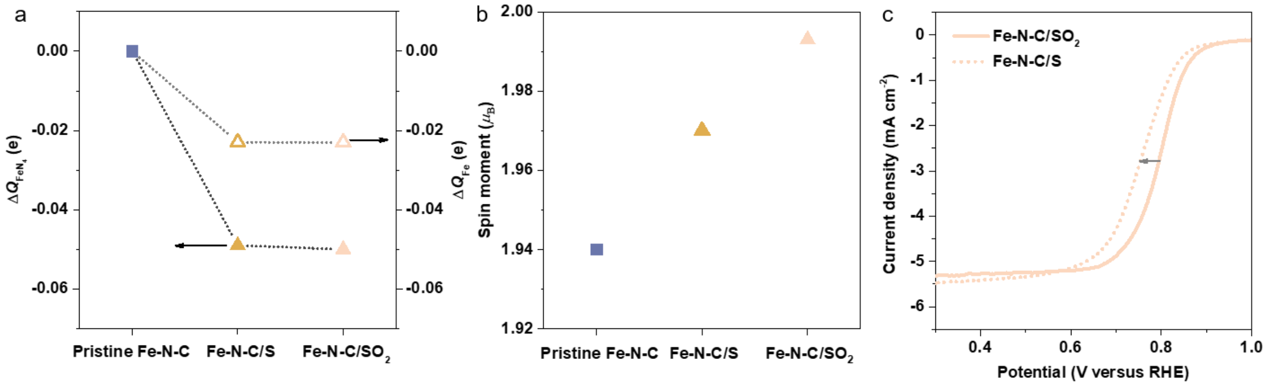

Figure S4. (a) Charge changes of Fe and FeN_4 of Fe-N-C/S and Fe-N-C/SO_2 catalysts with relative to those of pristine Fe-N-C catalysts. (b) Spin moment of Fe centers in pristine Fe-N-C, Fe-N-C/S and Fe-N-C/SO_2 catalysts. (c) ORR polarization curves of Fe-N-C/S and Fe-N-C/SO_2.

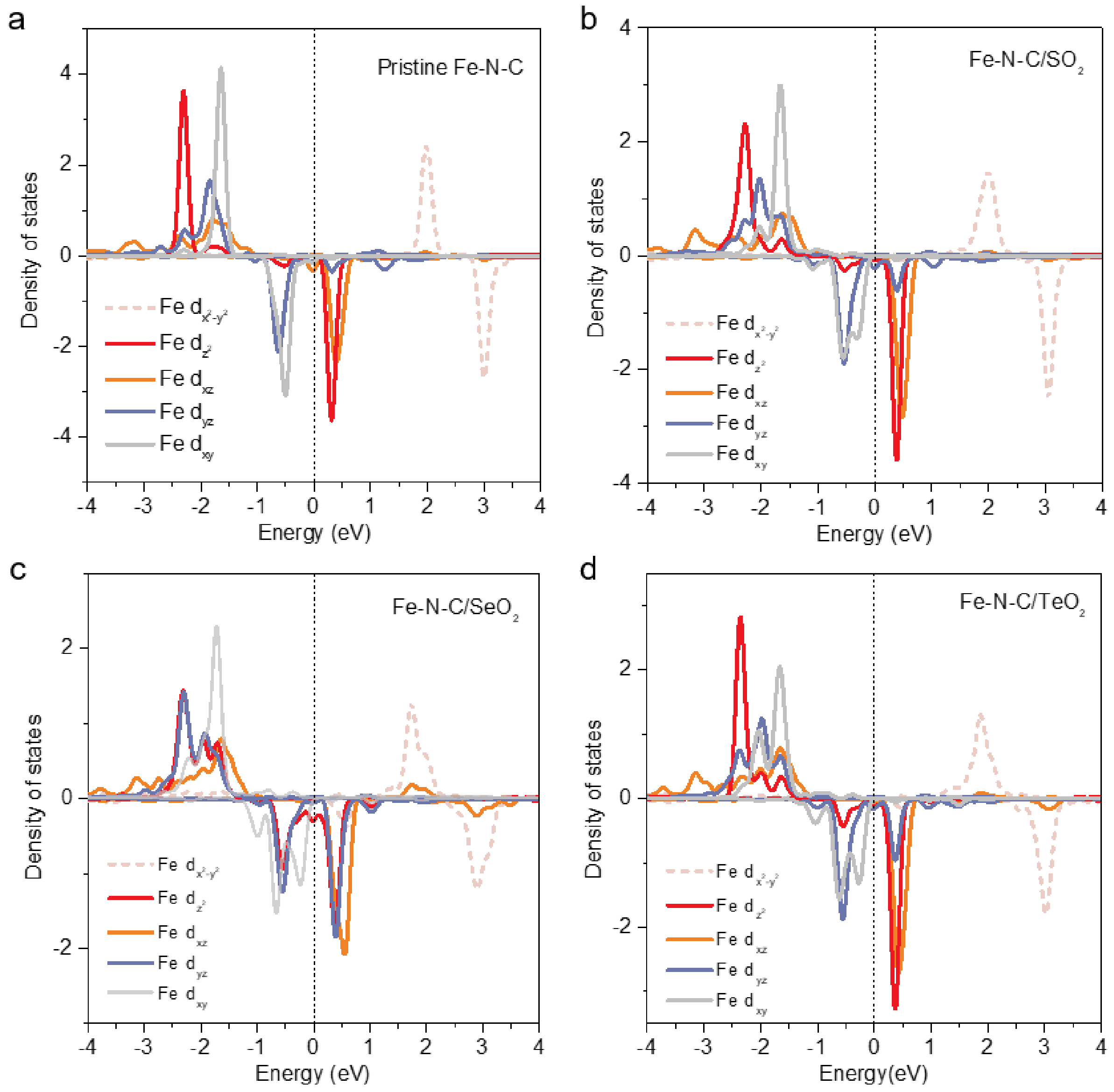

Figure S5. Projected density of states on Fe *d*-orbitals for (a) Pristine Fe-N-C, (b) Fe-N-C/SO_2, (c) Fe-N-C/SeO_2, and (d) Fe-N-C/TeO_2.

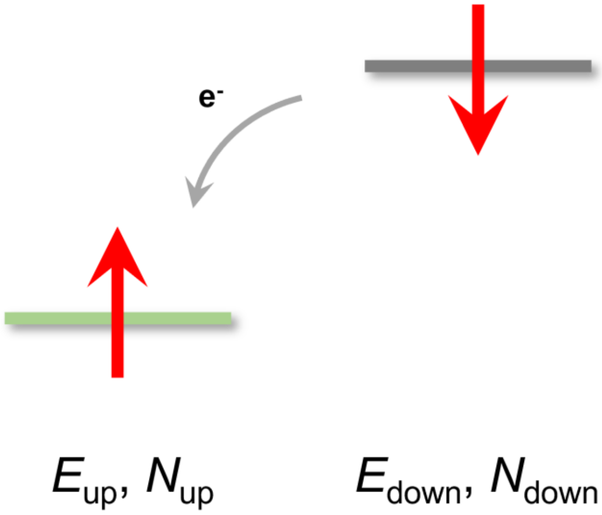

Figure S6. Energy level diagram of spin-up and spin-down Fe *d* orbitals in Fe-N-C catalysts.

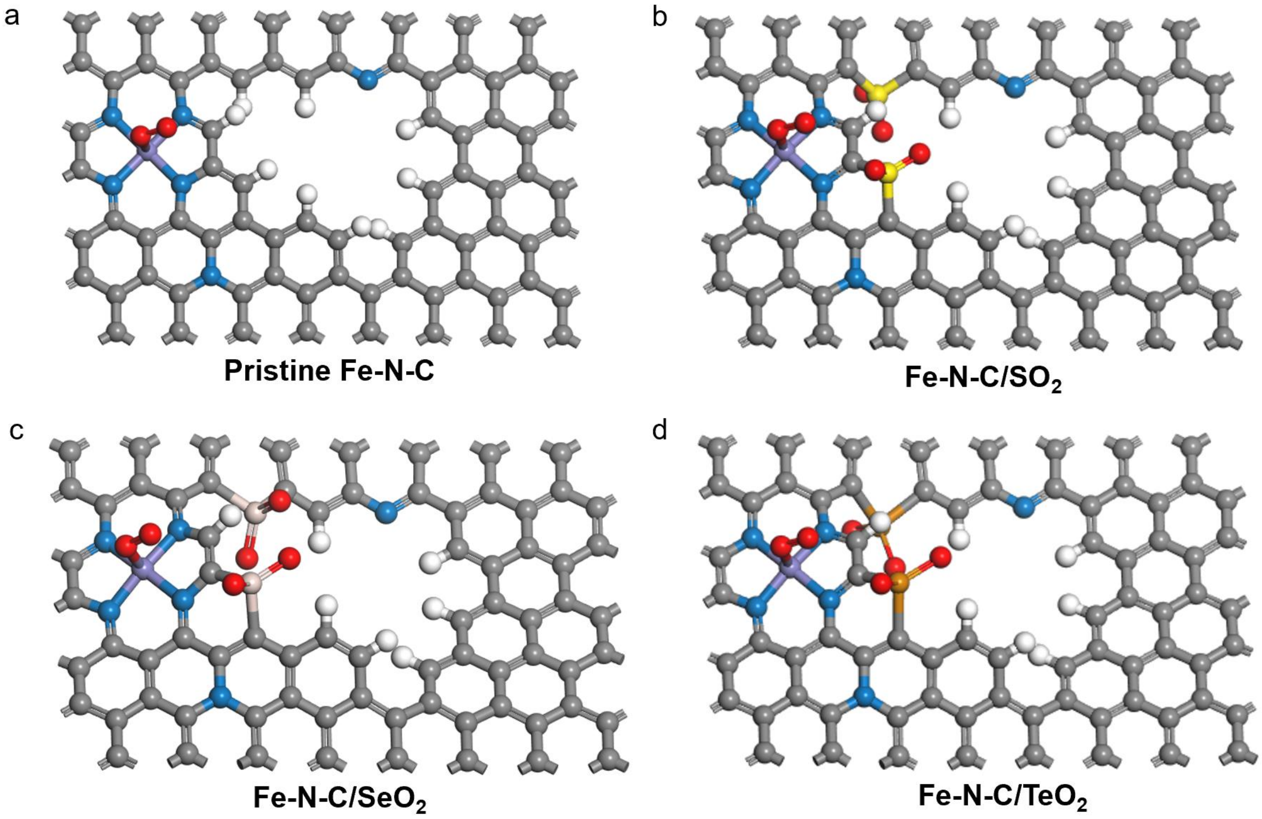

Figure S7. Optimized atomic structures of O_2 adsorbed on (a) pristine Fe-N-C, (b) Fe-N-C/SO_2, (c) Fe-N-C/SeO_2, and (d) Fe-N-C/TeO_2. Color codes are the same as in **Figure S1**. The adsorption free energies of O_2 (ΔG_{O_2*}) on Fe-N-C/XO_2 were shown in **Figure 2a**.

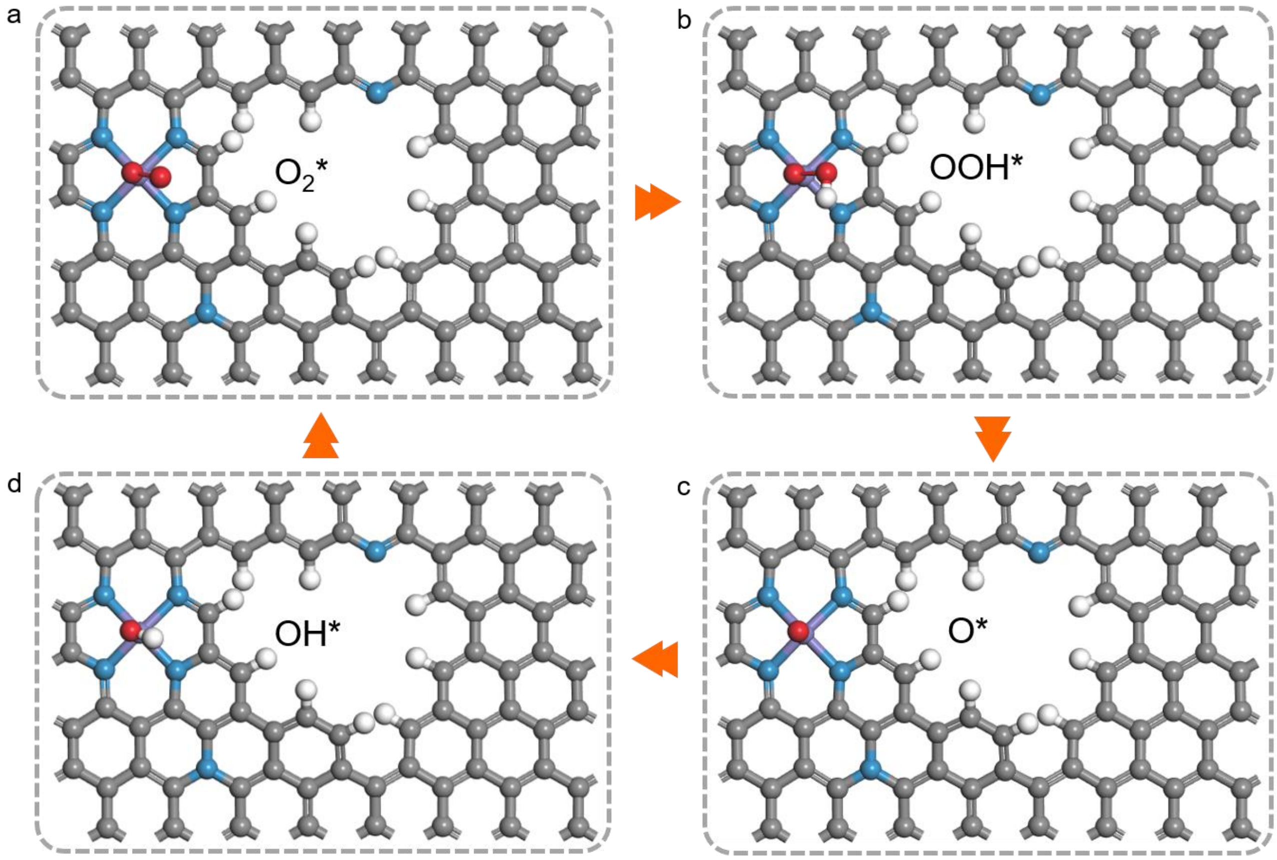

Figure S8. Optimized atomic structures of the ORR intermediates adsorbed on pristine Fe-N-C. (a) O_2*. (b) OOH*. (c) O*. (d) OH*. Color codes are the same as in **Figure S1**.

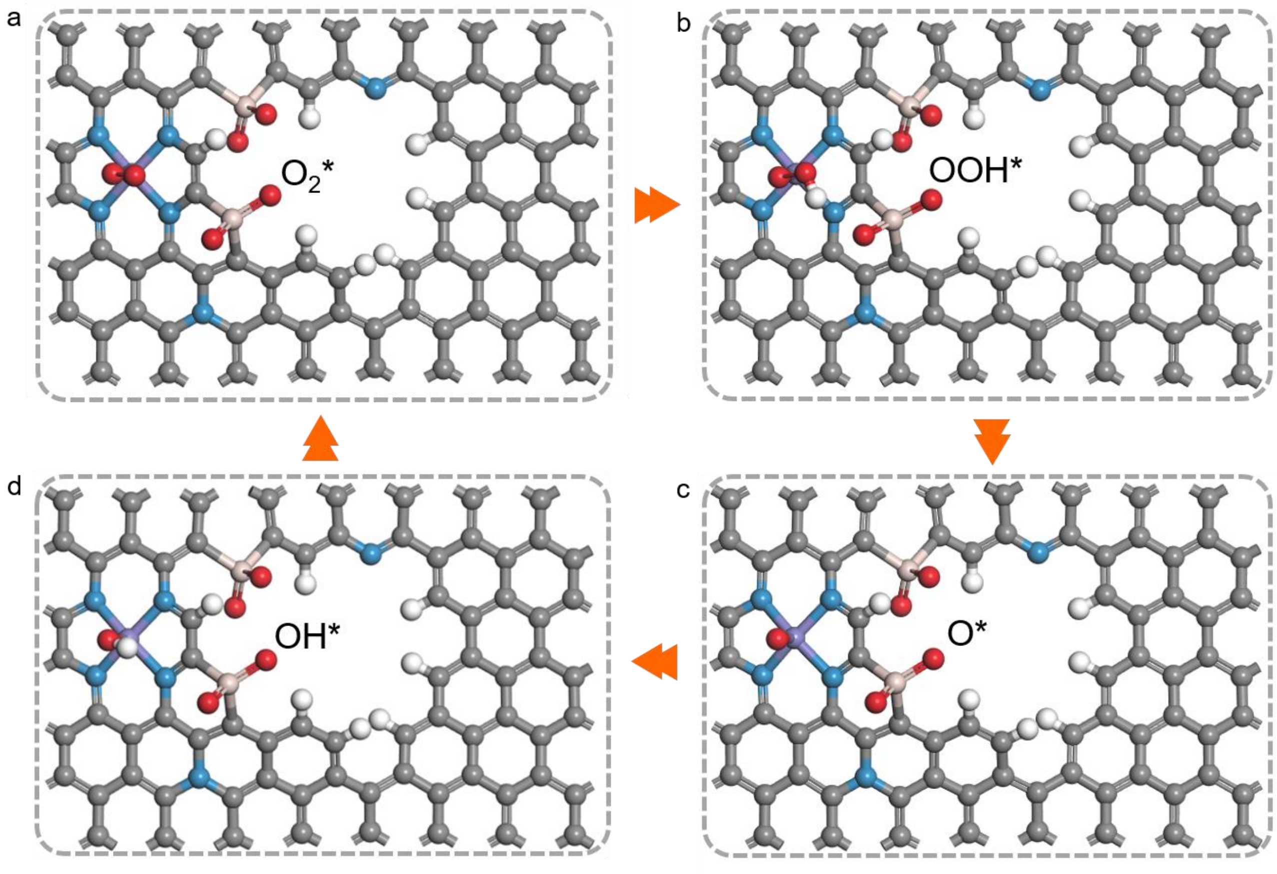

Figure S9. Optimized atomic structures of the ORR intermediates adsorbed on Fe-N-C/SeO_2. (a) O_2*. (b) OOH*. (c) O*. (d) OH*. Color codes are the same as in **Figure S1**.

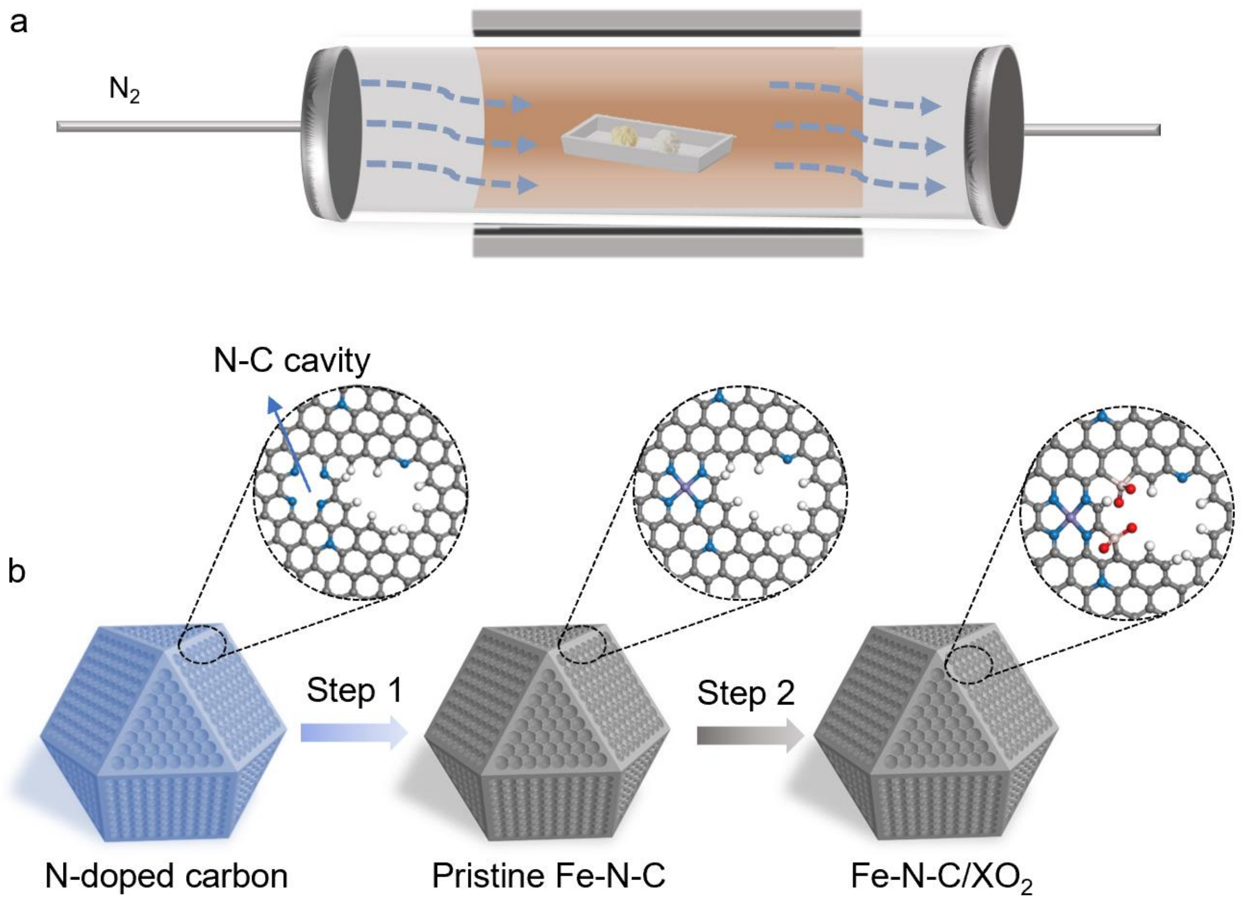

Figure S10. Schematic diagram of preparation process of Fe-N-C catalysts. (a) Apparatus diagram. (b) Synthetic process. In step 1, the N-doped carbon was placed in the centre of a quartz tube and anhydrous $FeCl_3$ powder was placed 2.5 cm upstream from the tube centre. In this step, gas-phase iron was captured by N-doped C to form Fe-N_4 moieties. In step 2, pristine Fe-N-C and X powder (X=S, Se, Te) were placed as in step 1 and then heated to the target temperature to incorporate the XO_2 groups in the carbon plane of Fe-N-C catalysts.

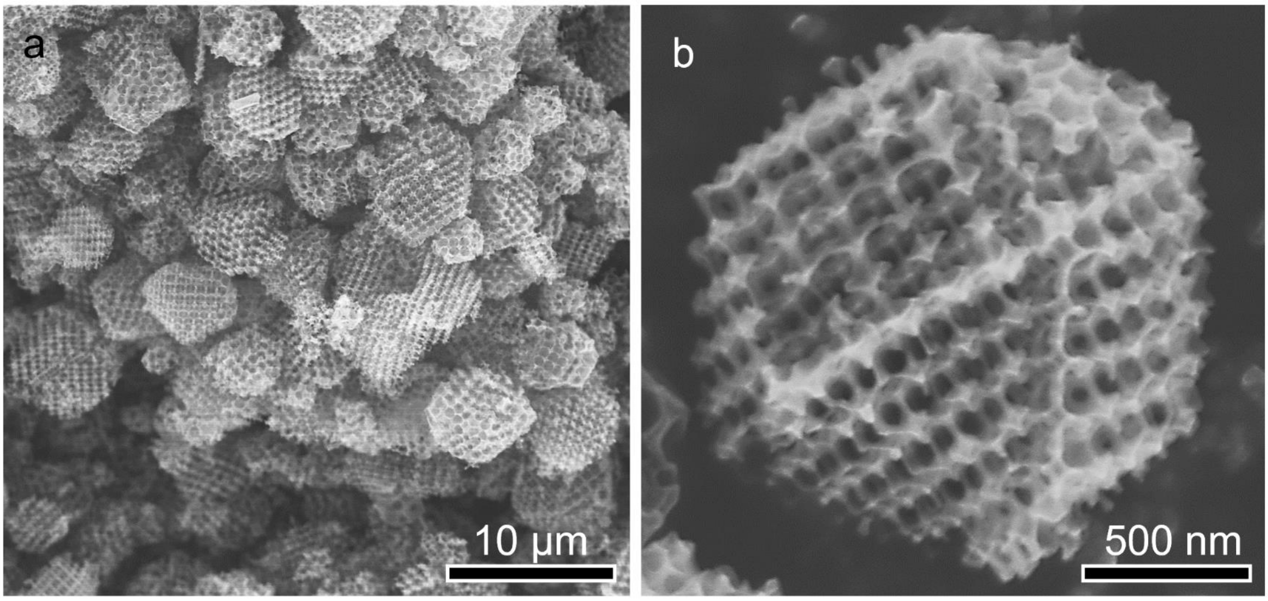

Figure S11. Characterization of pristine Fe-N-C catalyst. (a) Low and (b) high magnification SEM images.

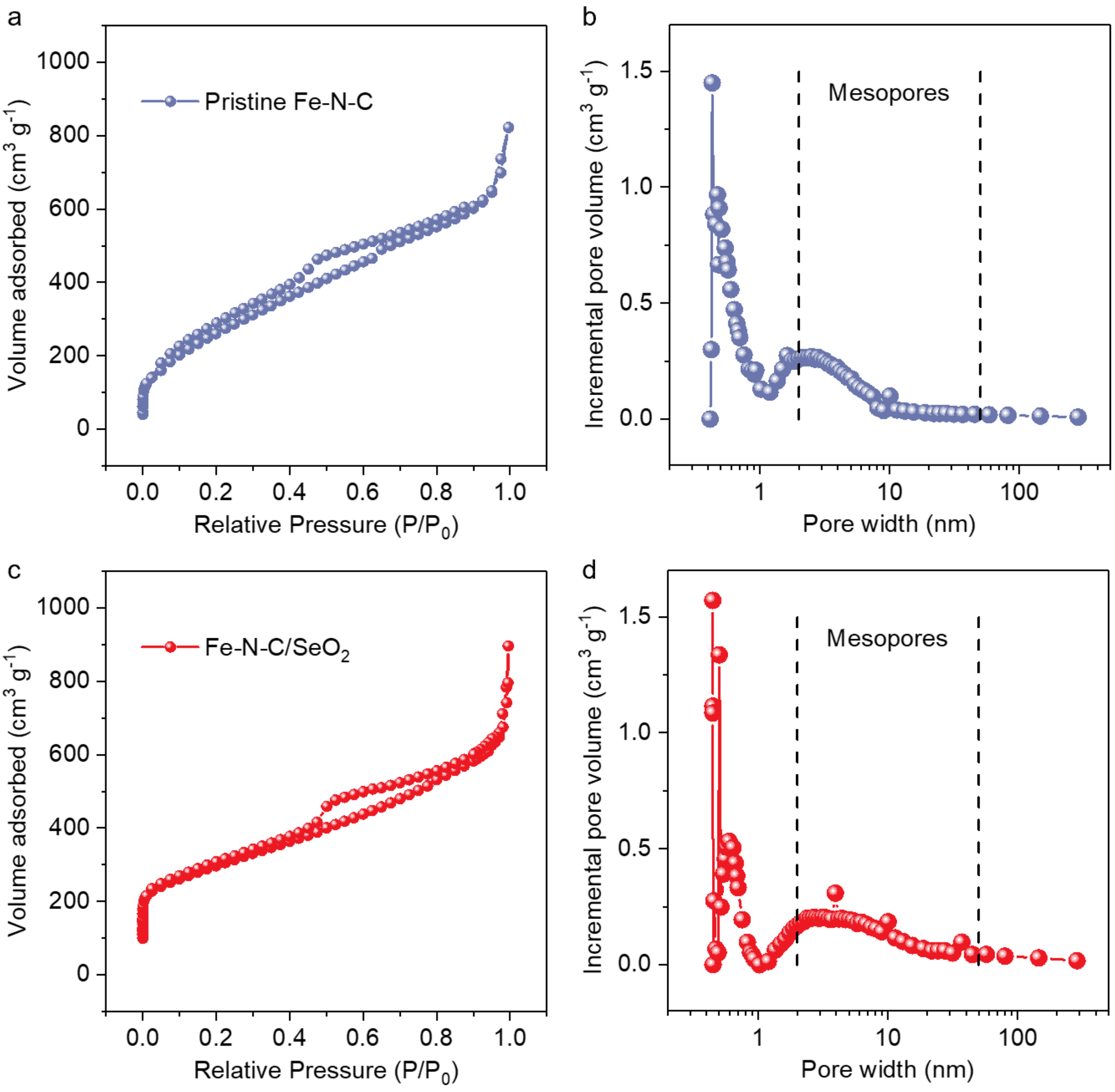

Figure S12. Characterization the surface area of pristine Fe-N-C and Fe-N-C/SeO_2. (a, c) N_2 sorption isotherms. (b, d) Pore size distribution. The remarkable hysteresis loop of type IV isotherms in (a, c) indicates that the dominant pores in Fe-N-C catalysts are mesopores. The Brunauer-Emmett-Teller surface area of pristine Fe-N-C and Fe-N-C/SeO_2 is 1051.2 m^2 g^{-1} and 1042.3 m^2 g^{-1}, respectively.

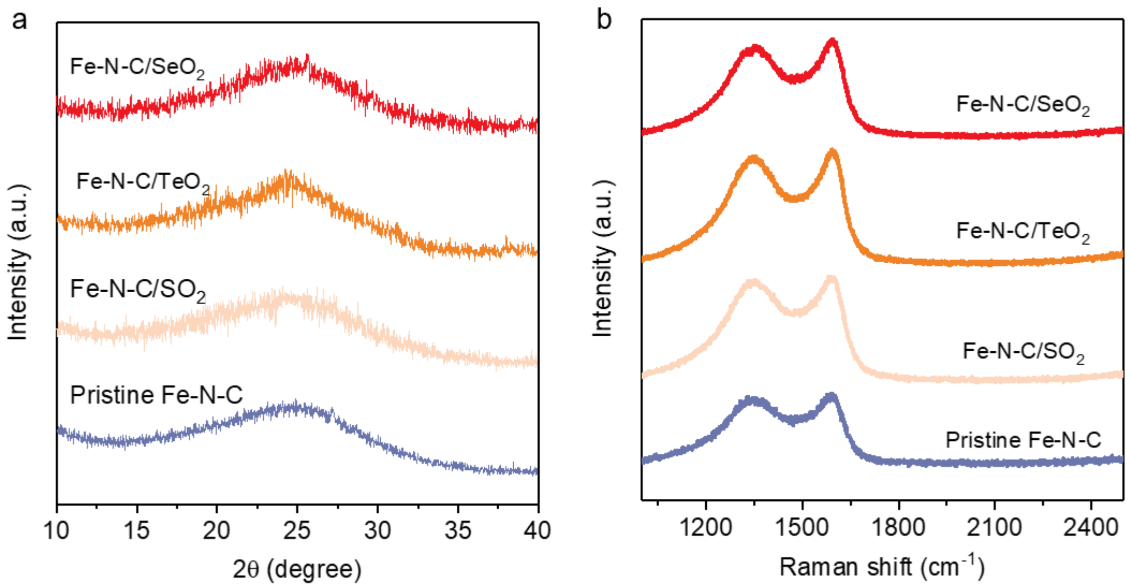

Figure S13. (a) XRD patterns and (b) Raman spectra of pristine Fe-N-C and Fe-N-C/XO_2. The identical XRD peak widths and intensity ratios between D band to G band (~1.14) indicate that the incorporation of XO_2 groups does not alter the carbon plane size of Fe-N-C catalysts.

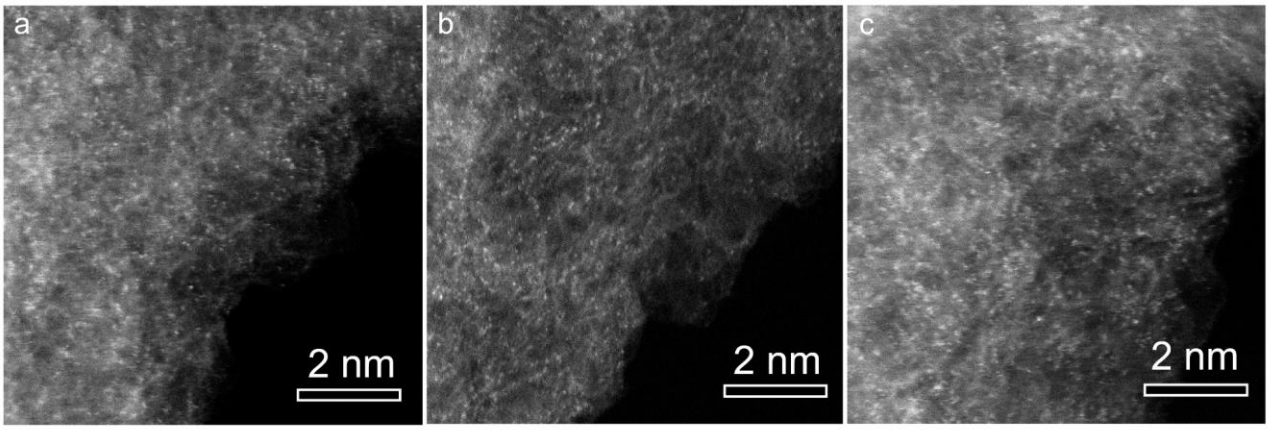

Figure S14. HADDF-STEM images of (a) pristine Fe-N-C, (b) Fe-N-C/SO_2 and (c) Fe-N-C/TeO_2.

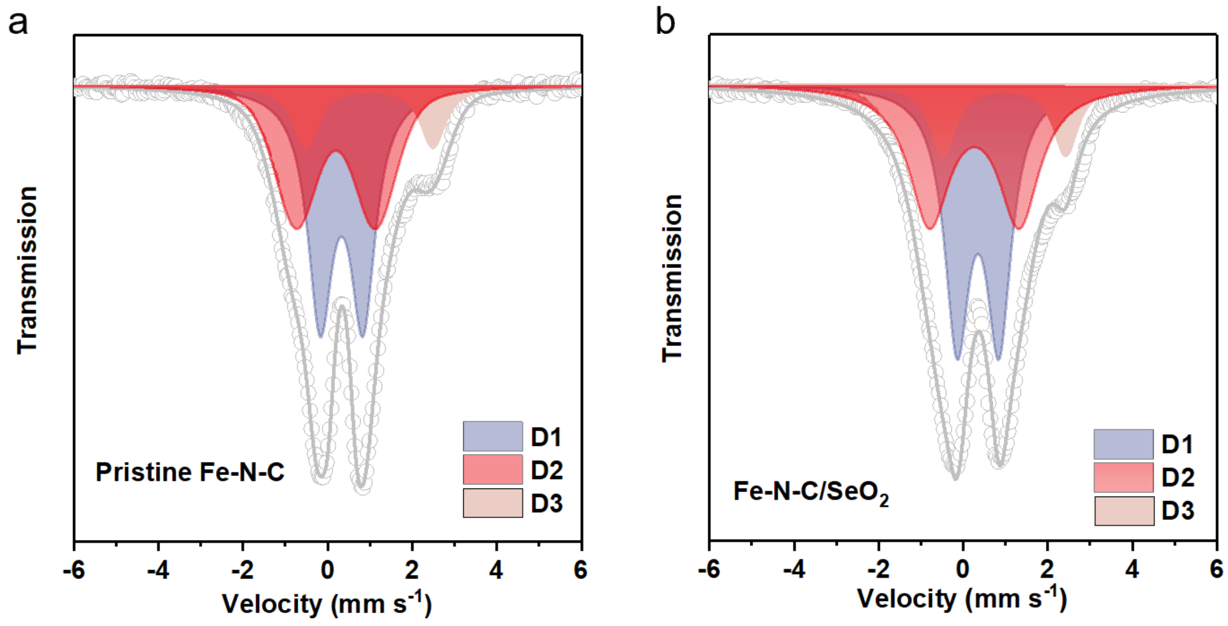

Figure S15. ^{57}Fe Mössbauer spectroscopic spectrum of pristine Fe-N-C and Fe-N-C/SeO_2. Note that D1, D2 and D3 signals in the Mössbauer spectra correspond to high-spin Fe^{3+}, intermedium-spin Fe^{2+} and high-spin Fe^{2+} in Fe-N-C catalysts, respectively[9, 10]. We note that the contents of D1, D2 and D3 in Fe-N-C and Fe-N-C/SeO_2 catalysts do not change significantly (**Table S3**). In our opinion, it is reasonable—our calculation and magnetic measurement results show that the Fe centers in our Fe-N-C catalysts are mainly in intermedium-spin state and the distortion of FeN_4 changes the Fe spin moments, but not the Fe spin-state.

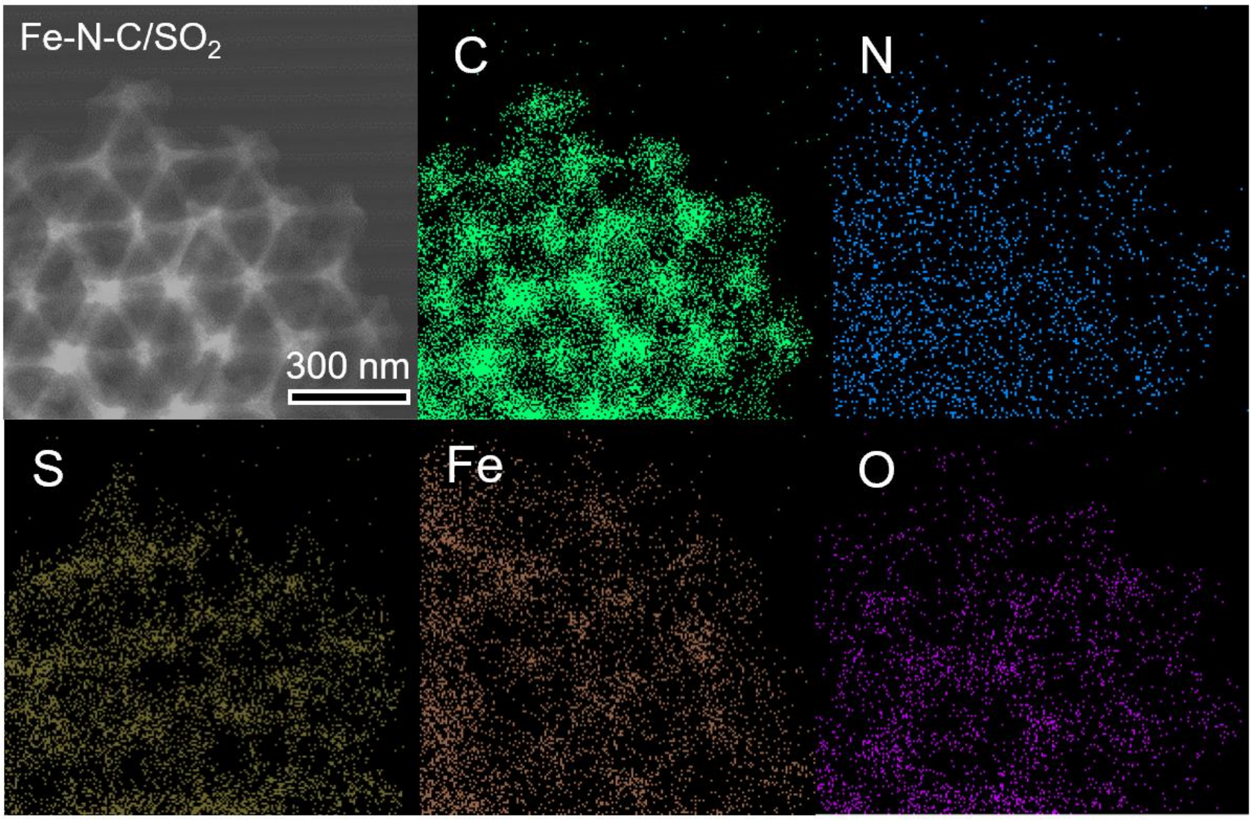

Figure S16. TEM image of Fe-N-C/SO_2 and the corresponding EDS mappings of C, N, S, Fe and O elements.

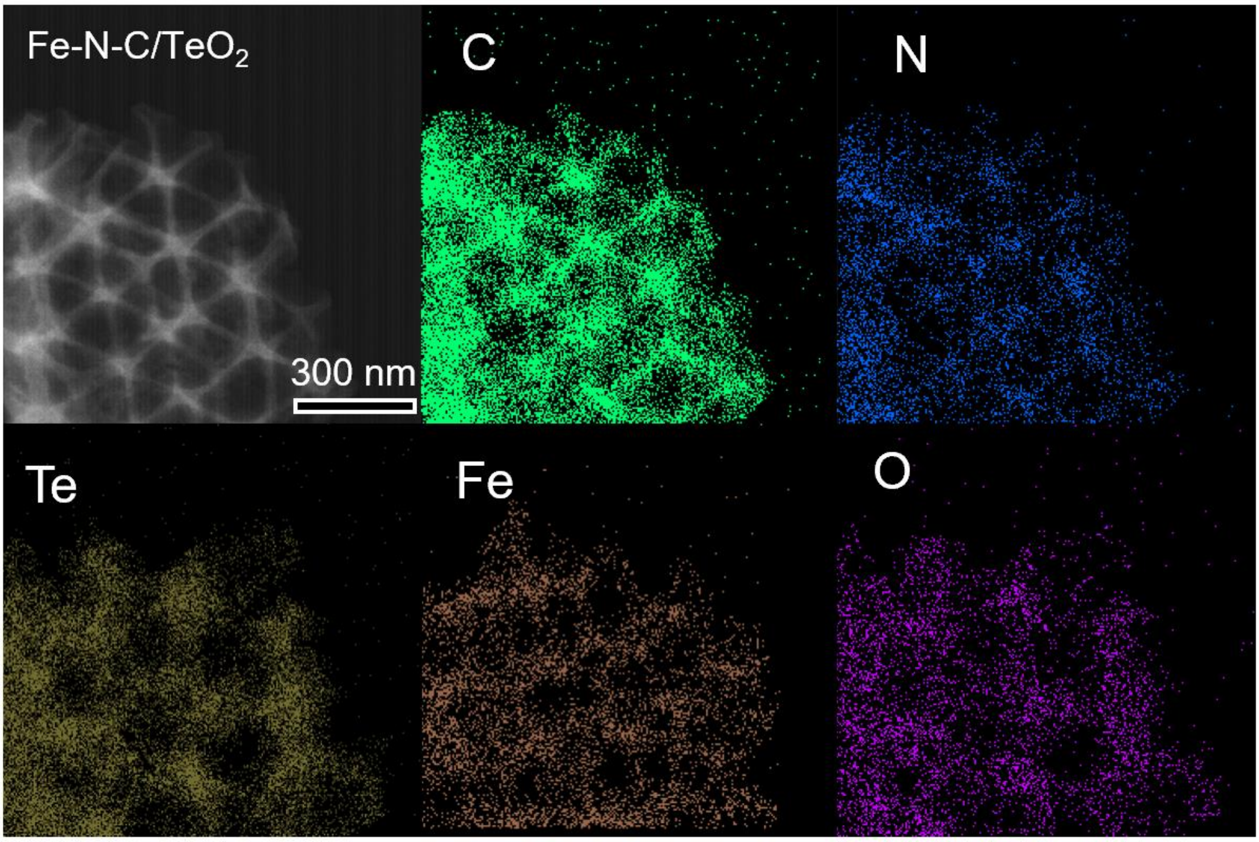

Figure S17. TEM image of Fe-N-C/TeO_2 and the corresponding EDS mappings of C, N, Te, Fe and O elements.

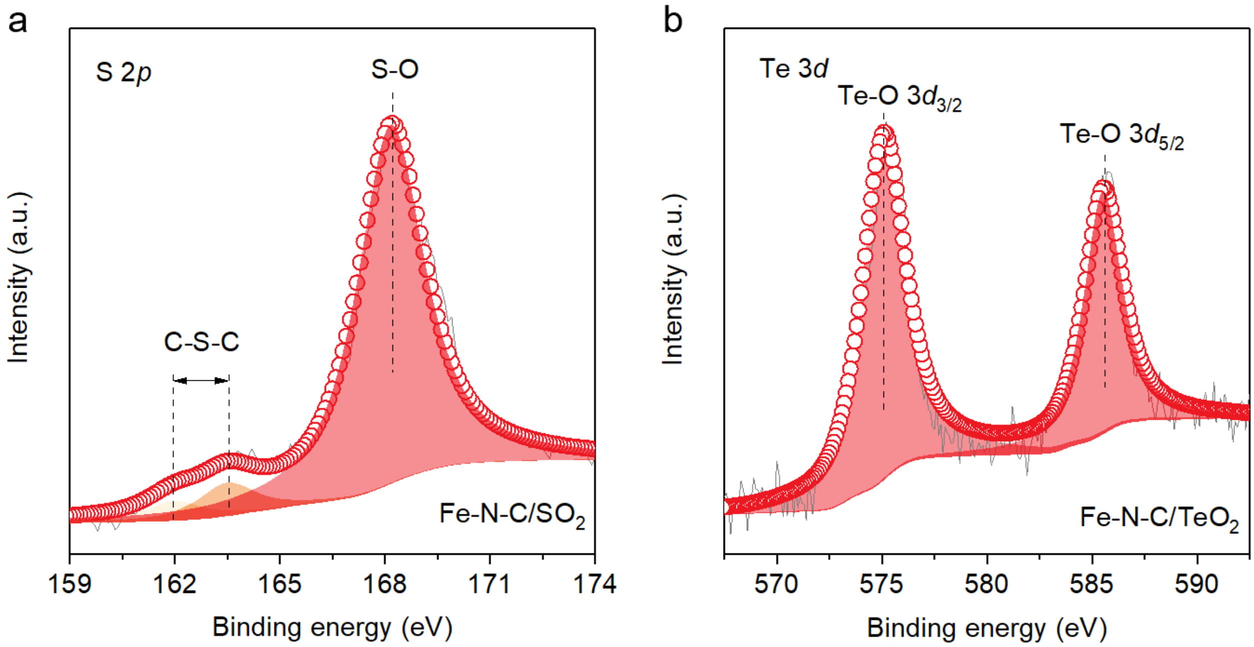

Figure S18. (a) S 2*p* XPS spectrum of Fe-N-C/SO_2. (b) Te 3*d* XPS spectrum of Fe-N-C/TeO_2. As shown in this figure and **Figure 3f**, almost all the X added to the carbon matrix is oxidized to XO_2. The contents of XO_2 in the Fe-N-C catalysts were provided in **Table S4**.

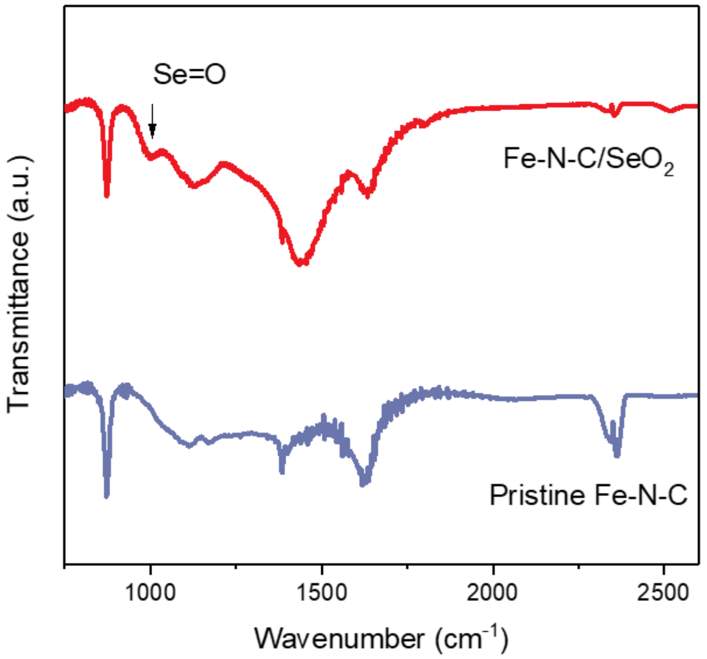

Figure S19. FTIR spectra of pristine Fe-N-C and Fe-N-C/SeO_2. For Fe-N-C/SeO_2, the strong bands at 900-1100 cm^{-1} (Se=O stretch)[11, 12] demonstrate the existence of SeO_2 groups.

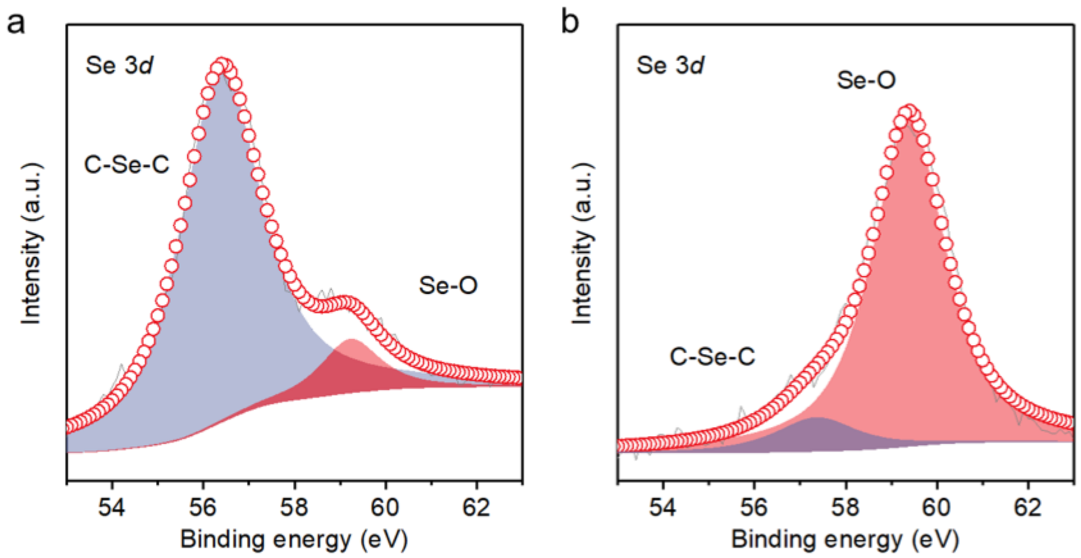

Figure S20. Se 3*d* XPS spectra of Fe-N-C/SeO_2 (a) before and (b) after Se oxidation, respectively. As shown, C-Se-C is dominant in the pre-introduced Se doped Fe-N-C, suggesting that the incorporated Se atoms in Fe-N-C catalysts should distribute at the edge of carbon. It is reasonable that Se is the sixth main group element and can form covalent bonds with two C atoms. Obviously, after being oxidized, XO_2 groups should be also at the edge of carbon.

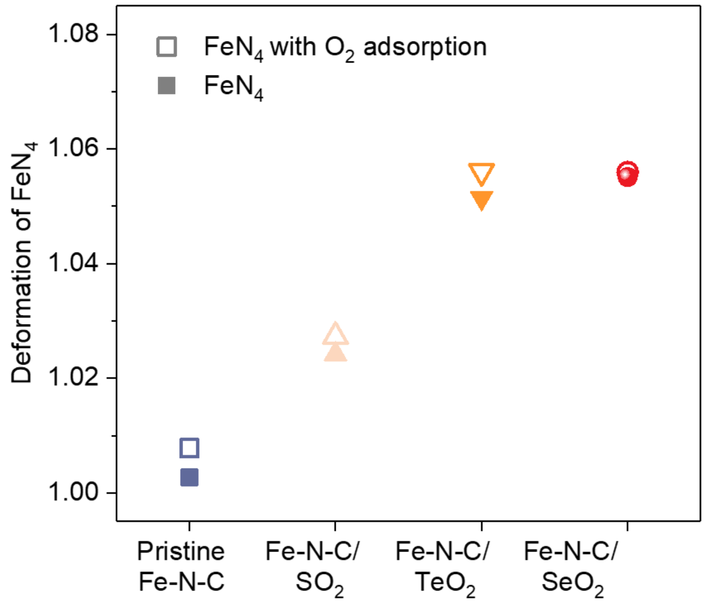

Figure S21. Deformation values of FeN_4 for pristine Fe-N-C and Fe-N-C/XO_2 catalysts with or without O_2 adsorption.

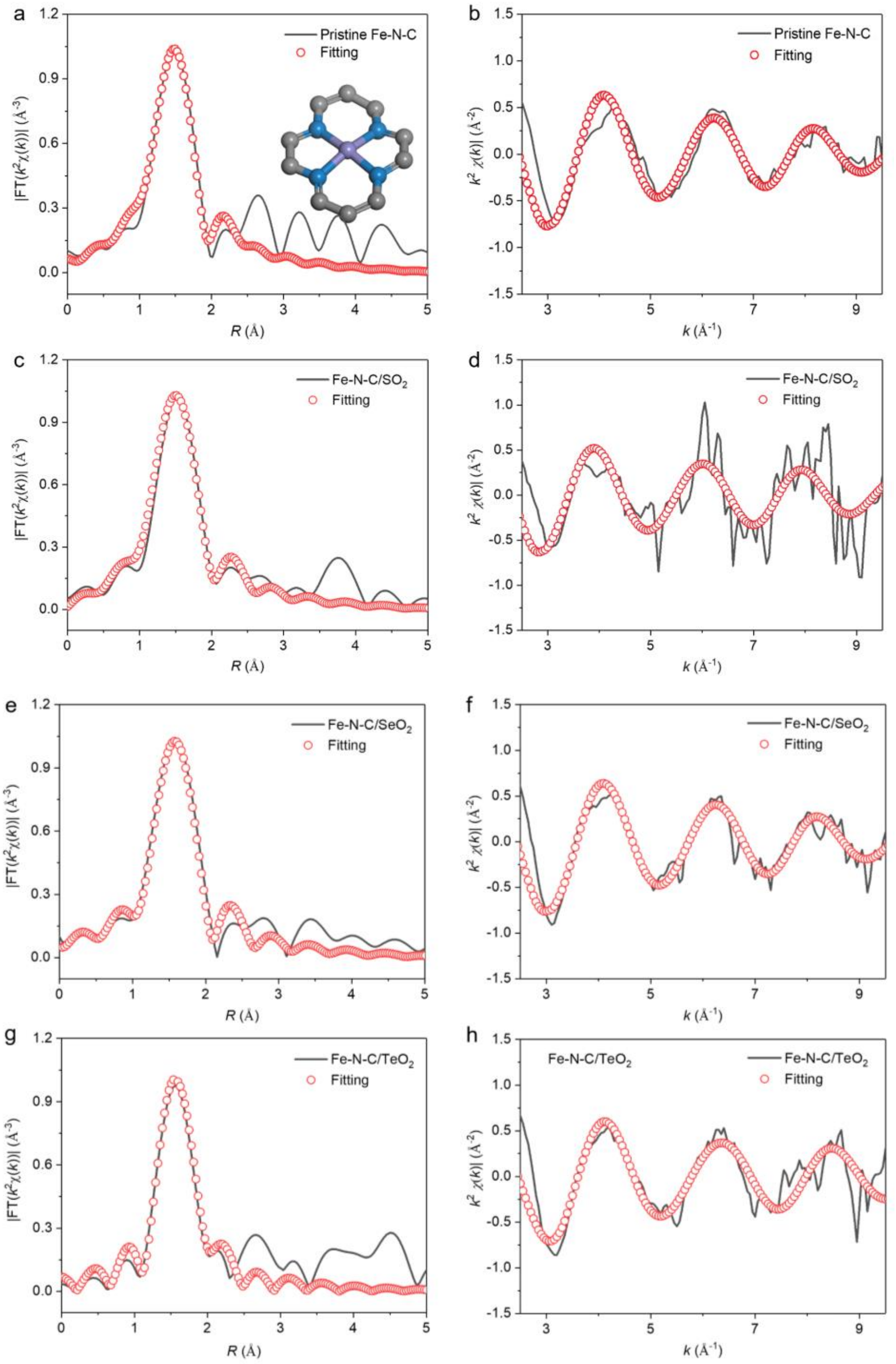

Figure S22. Experimental EXAFS and corresponding fitting curves of Fe-N-C catalysts. (a) and (b) Pristine Fe-N-C. (c) and (d) Fe-N-C/SO_2. (e) and (f) Fe-N-C/SeO_2. (g) and (h) Fe-N-C/TeO_2. (a), (c), (e) and (g) present curves in *R* space; (b), (d), (f) and (h) present curves in *K* space.

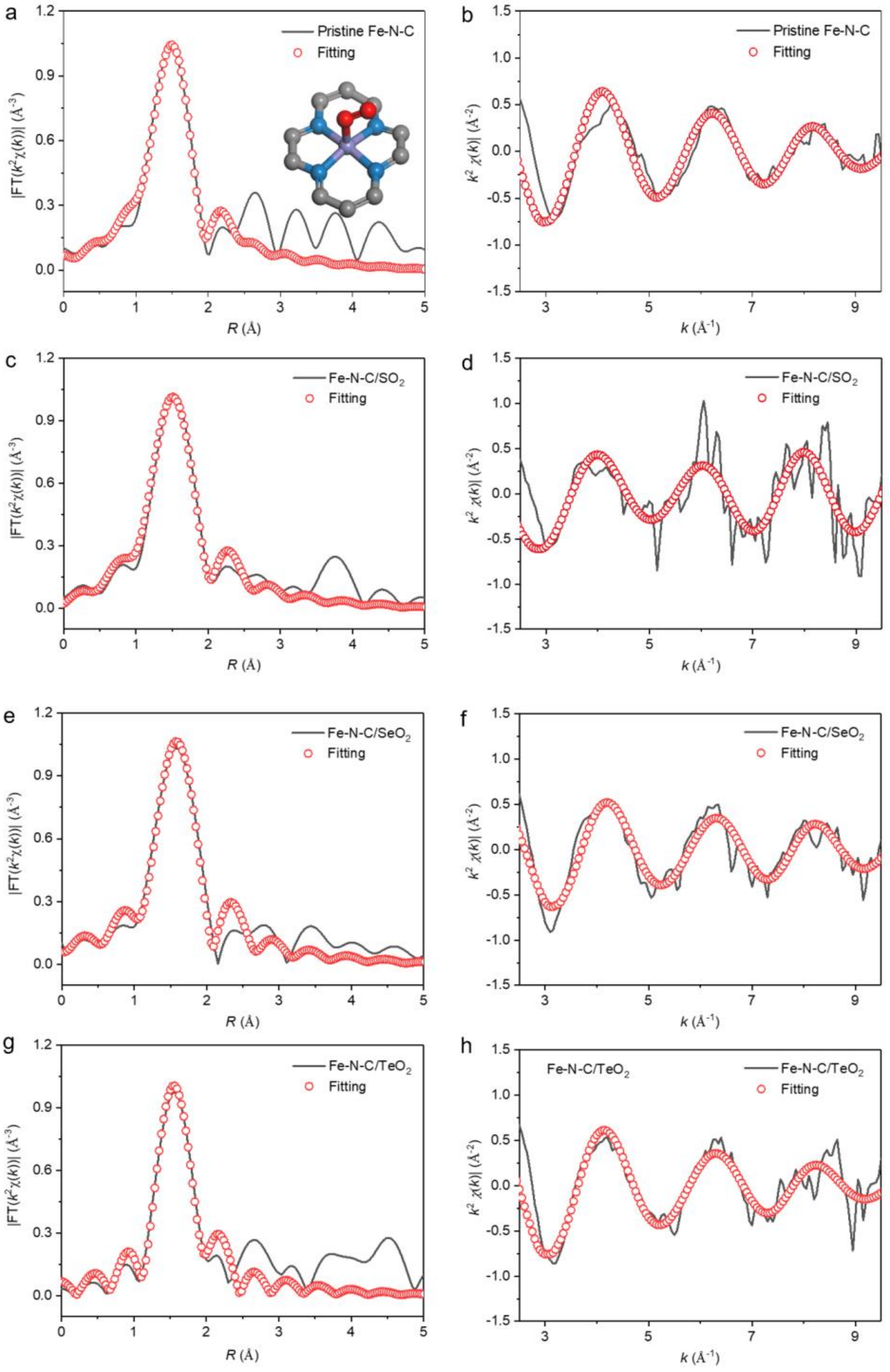

Figure S23. Experimental EXAFS and corresponding fitting curves of Fe-N-C catalysts with O_2 adsorption model. (a) and (b) Pristine Fe-N-C. (c) and (d) Fe-N-C/SO_2. (e) and (f) Fe-N-C/SeO_2. (g) and (h) Fe-N-C/TeO_2. (a), (c), (e) and (g) present curves in *R* space; (b), (d), (f) and (h) present curves in *K* space.

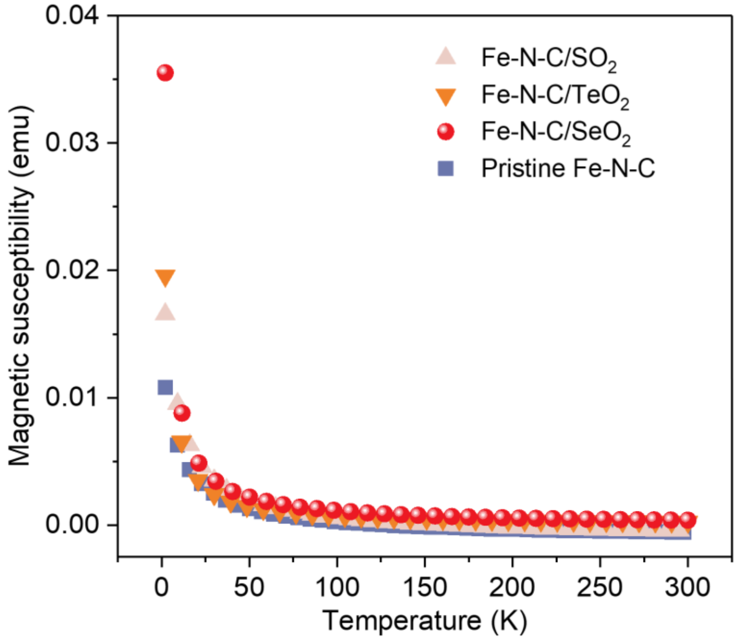

Figure S24. Magnetic susceptibility of Fe-N-C catalysts as a function of temperature.

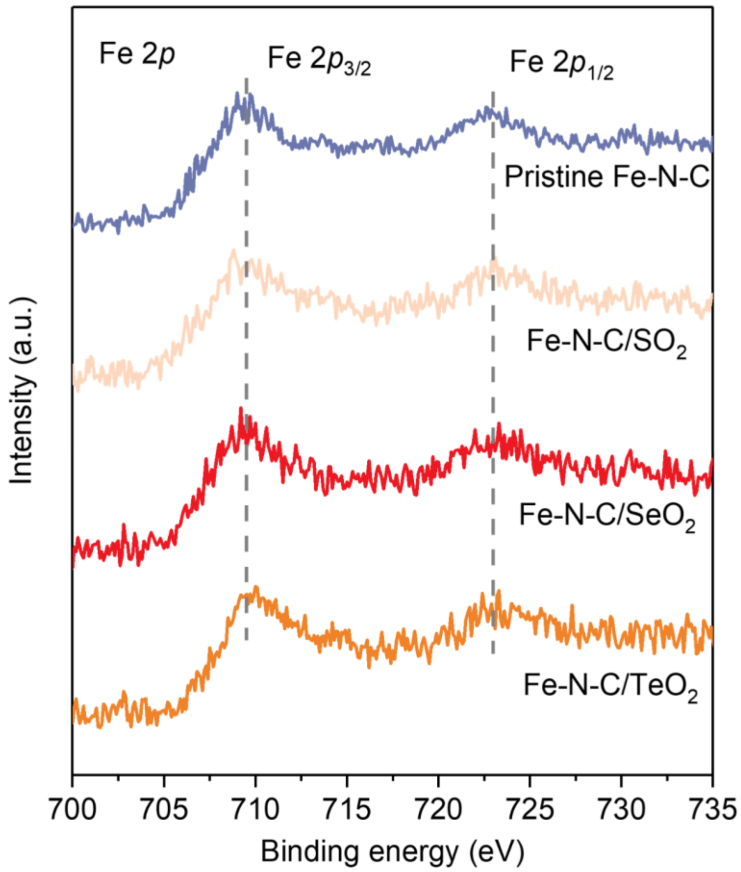

Figure S25. High-resolution XPS Fe 2*p* spectra of Fe-N-C catalysts. As shown, the Fe $2p_{3/2}$ and Fe $2p_{1/2}$ peaks in the spectra of pristine Fe-N-C and Fe-N-C/XO_2 are centered at 709.2 eV and 722.8 eV, which are assigned to Fe^{2+} (709.4-710.8 eV for Fe $2p_{3/2}$, 723.0-724.4 eV for Fe $2p_{1/2}$),[13, 14] indicating that these samples mainly contain ferrous species.

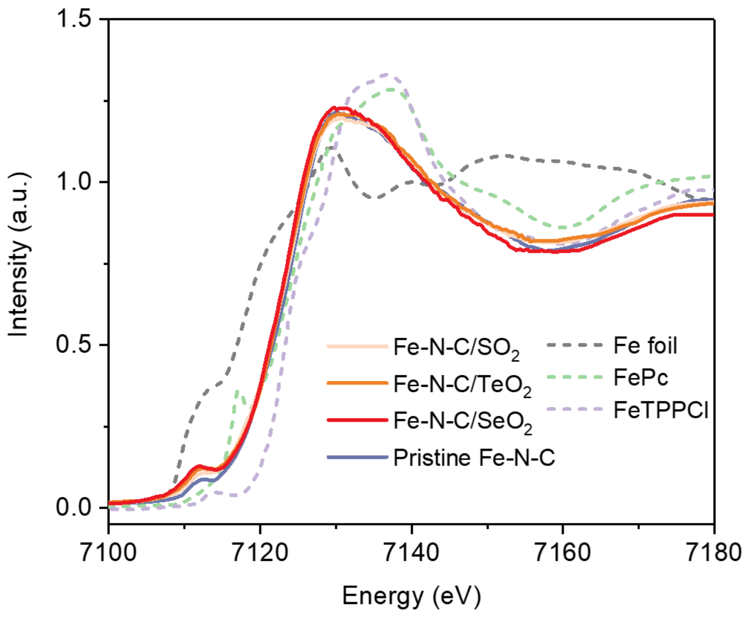

Figure S26. Fe K-edge XANES spectra of Fe-N-C catalysts and reference samples. The absorption threshold position of catalysts is close to that of phthalocyanine (FePc), implying that the chemical valence of iron is around +2, consistent with the XPS results.

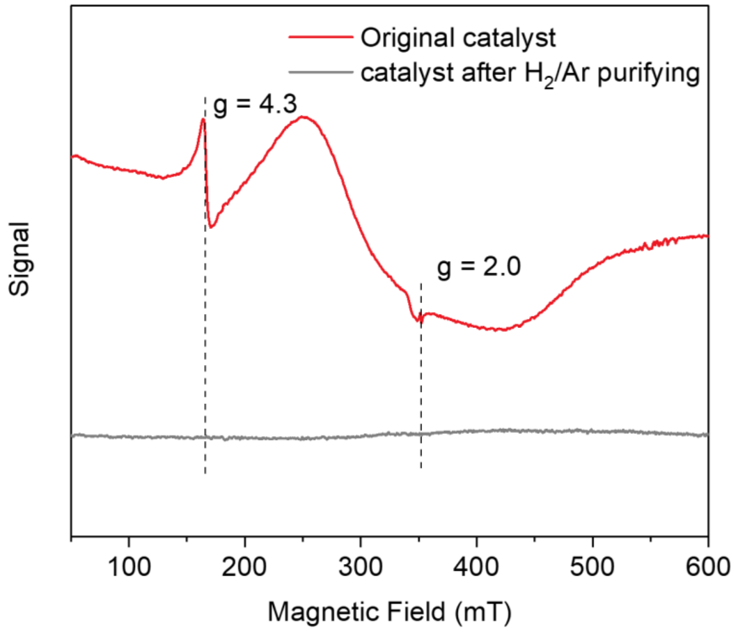

Figure S27. X-band EPR spectra of Fe-N-C/SeO_2 with and without H_2/Ar gas purifying. Microwave frequency 9.56 GHz, amplitude 0.3 mT, T = 5 K. Note that the signals at g = 4.3 and g = 2 are assigned to Fe^{3+} at the spin state of 5/2,[15] which disappears after H_2/Ar purification. This demonstrates that there is no Fe^{3+} in the catalyst after H_2/Ar purification.

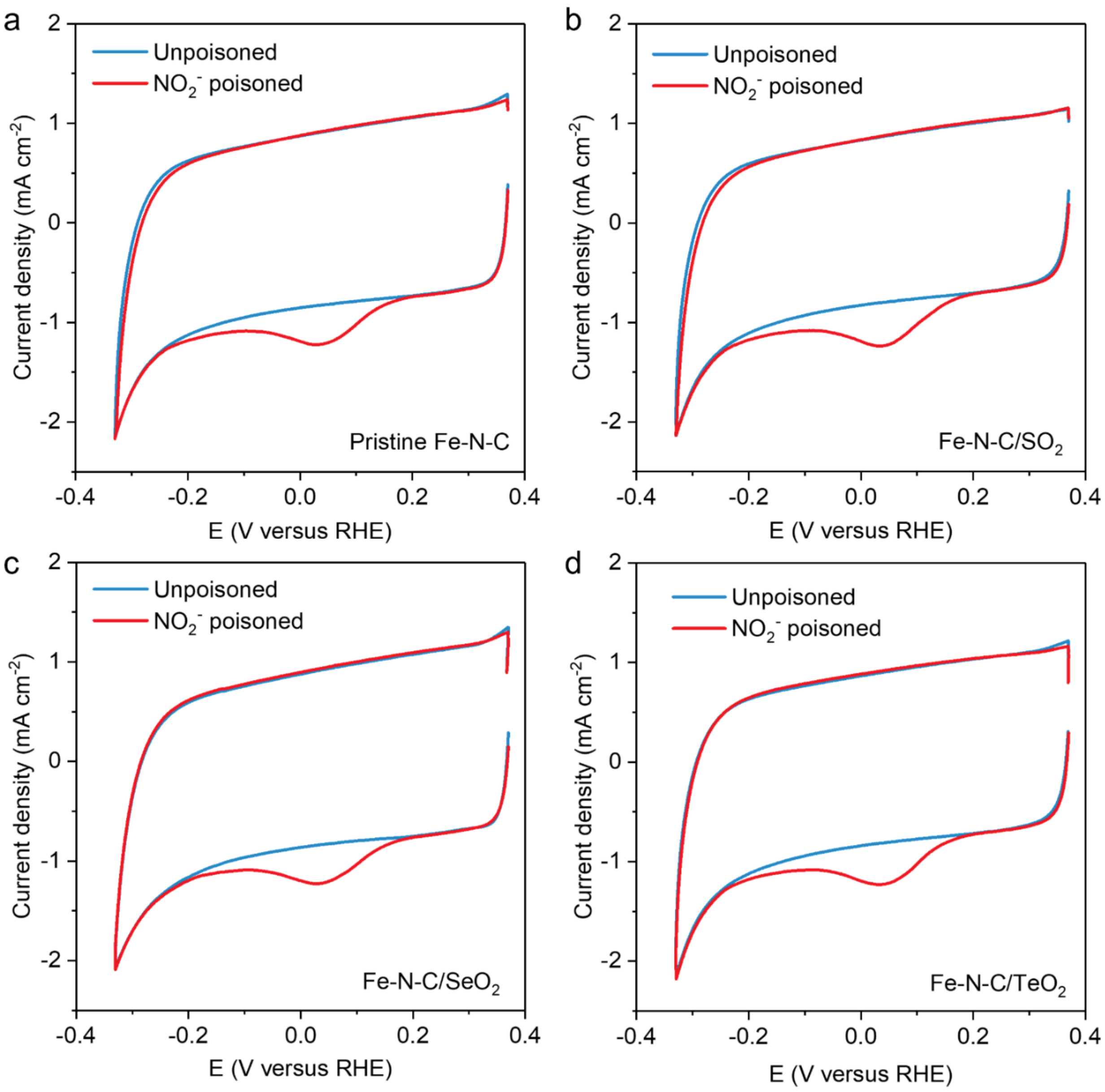

Figure S28. Nitrite stripping voltammetry in N_2-saturated 0.5 M acetate electrolyte buffer (pH 5.2) with a catalyst loading of 0.27 mg cm^{-2} and a scan rate of 10 mV s^{-1}. (a) Pristine Fe-N-C. (b) Fe-N-C/SO_2. (c) Fe-N-C/SeO_2. (d) Fe-N-C/TeO_2. The numbers of FeN_4 active sites in these catalysts determined from the reversible nitrite poisoning experiment were summarized in **Table S7**.

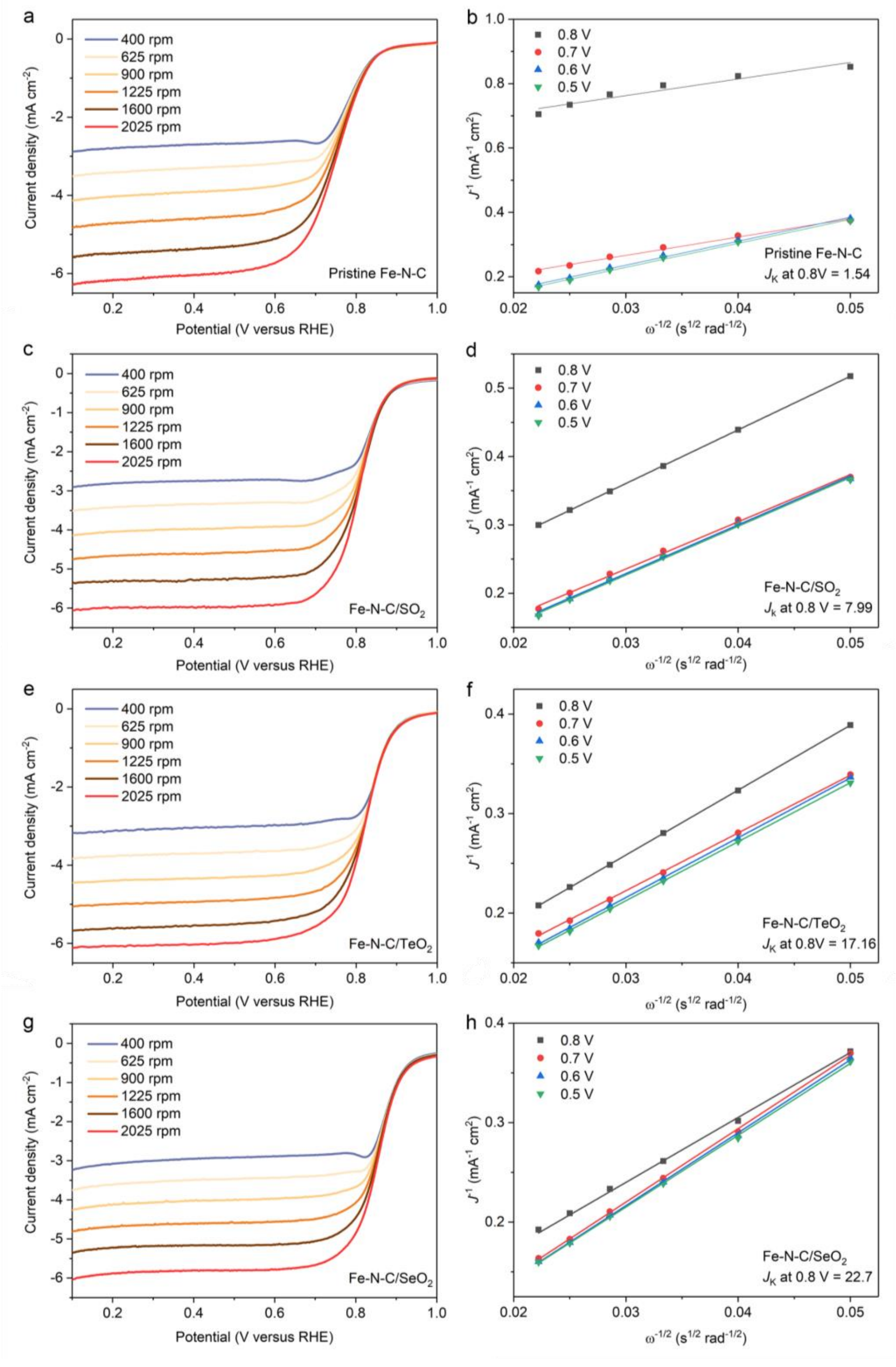

Figure S29. LSV curves of pristine Fe-N-C and Fe-N-C/XO_2 at varied rotation rates and their corresponding Koutecky-Levich plots. (a, b) Pristine Fe-N-C. (c, d) Fe-N-C/SO_2. (e, f) Fe-N-C/SeO_2. (g, h) Fe-N-C/TeO_2.

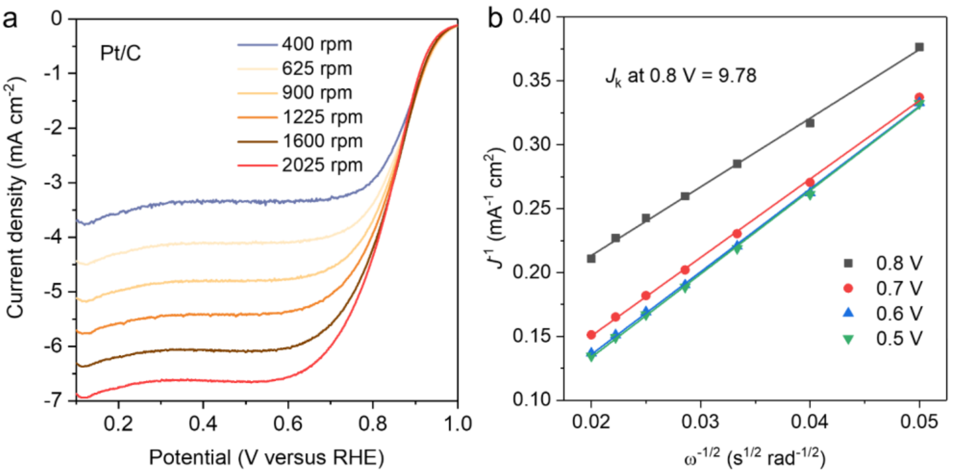

Figure S30. (a) LSV curves of Pt/C at various rotation rates and (b) corresponding Koutecky-Levich plot.

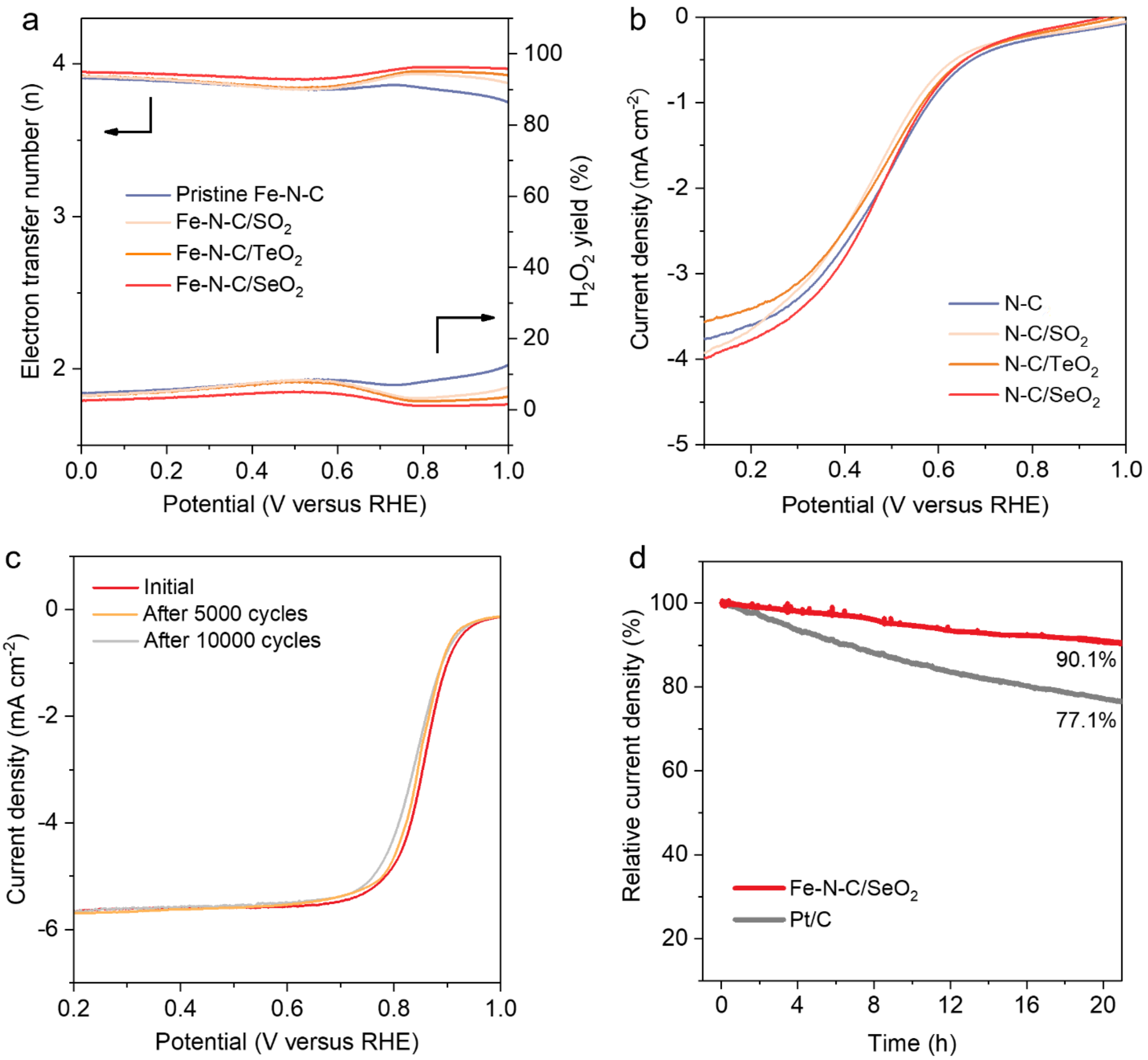

Figure S31. (a) Electron transfer number (*n*) and H_2O_2 selectivity of pristine Fe-N-C and Fe-N-C/XO_2 determined by RRDE tests. (b) ORR linear sweep polarization curves for N-C and N-C/XO_2 in O_2-saturated 0.5 M H_2SO_4 electrolyte. (c) ORR polarization curves of Fe-N-C/SeO_2 before and after 5,000 and 10,000 cyclic voltammetry cycles, assessed by cycling the catalyst between 1.1 and 0.2 V versus RHE at 50 mV s^{-1}. (d) Current-time responses of Fe-N-C/SeO_2 and Pt/C in O_2-saturated 0.50 M H_2SO_4 and 0.10 M $HClO_4$, respectively, at a potential of 0.6 V versus RHE.

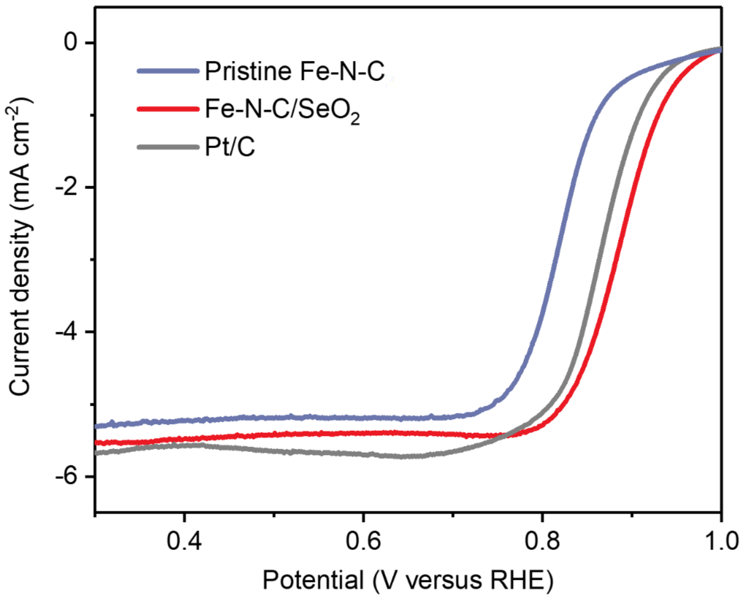

Figure S32. Linear scan voltammogram (LSV) curves of Fe-N-C catalysts and Pt/C catalyst in O_2-saturated 0.10 M KOH at 1,600 rpm with a scan rate of 10 mV s^{-1}.

Supplementary Tables

Table S1. The parameters to estimate the deformation of FeN_4 (D_{FeN4}).

Sample	d_1	d_2	d_3	d_4	α_1	α_2	α_3	α_4	D_d[a]	D_α[b]	$d_{average}$[c]
Pristine Fe-N-C	1.921	1.914	1.901	1.912	90.922	89.822	90.505	88.536	1.002121	1.000597	1.912
Fe-N-C/SO_2	1.913	1.922	1.891	1.926	91.469	88.557	90.605	89.050	1.023300	1.000886	1.913
Fe-N-C/SeO_2	1.905	1.934	1.891	1.956	91.852	87.300	90.383	88.764	1.050119	1.004747	1.922
Fe-N-C/TeO_2	1.902	1.931	1.886	1.951	92.410	86.985	90.652	89.533	1.050237	1.001168	1.918

[a]$D_d = d_2 \times d_4/(d_1 \times d_3)$; [b]$D_\alpha = 360/(\alpha_1+\alpha_2+\alpha_3+\alpha_4)$; [c]$d_{average} = (d_1+d_2+d_3+d_4)/4$. Note that the definition of d_1-d_4 and α_1-α_4 is provided in the inset of **Figure 1d** and **Figure S2a**.

Table S2. The calculated Fe 3*d* electron-filling in pristine Fe-N-C and Fe-N-C/XO_2.

Sample	Electron description	d_{xy}	d_{yz}	d_{xz}	d_{z^2}	$d_{x^2-y^2}$
Pristine Fe-N-C	Spin up	0.9251	0.8821	0.9147	0.8943	0.4516
	Spin down	0.8827	0.6657	0.1696	0.1521	0.3640
Fe-N-C/SO_2	Spin up	0.9249	0.8933	0.9164	0.9005	0.4613
	Spin down	0.8837	0.6323	0.1165	0.1744	0.3625
Fe-N-C/SeO_2	Spin up	0.9170	0.9045	0.8703	0.8984	0.5090
	Spin down	0.8675	0.4173	0.1353	0.4081	0.3366
Fe-N-C/TeO_2	Spin up	0.9201	0.8977	0.9061	0.9010	0.4746
	Spin down	0.8715	0.5917	0.1214	0.2239	0.3574

Table S3. ^{57}Fe Mössbauer parameters derived from the fittings. Isomer shift (IS), quadrupole splitting (QS), line width (LW), and relative spectral area % of each component.

Sample	Component	IS (mm s^{-1})	QS (mm s^{-1})	LW (mm s^{-1})	Area %
	D1	0.34	0.91	0.65	46
Pristine Fe-N-C	D2	0.40	2.51	0.90	42
	D3	0.77	2.60	0.97	12
	D1	0.37	0.97	0.59	45
Fe-N-C/SeO_2	D2	0.40	2.66	0.90	43
	D3	0.77	2.59	0.58	12

Table S4. Analysis of the contents of XO_2 in Fe-N-C catalysts by XPS.

Sample	C (at%)	N (at%)	O (at%)	X (at%)	Fe (at %)
Pristine Fe-N-C	86.99	7.84	4.66	-	0.51
Fe-N-C/SO_2	86.70	7.45	3.85	1.53	0.47
Fe-N-C/SeO_2	86.29	7.72	3.94	1.55	0.50
Fe-N-C/TeO_2	86.43	7.54	3.97	1.58	0.48

Note that X stands for S, Se and Te element in Fe-N-C/SO_2, Fe-N-C/SeO_2 and Fe-N-C/TeO_2, respectively.

Table S5. Fitting parameters of Fe *K*-edge EXAFS curves of pristine Fe-N-C and Fe-N-C/XO_2.

Sample	Path	N^a	R (Å)^b	σ^2 (× 10^{-3} Å^2)^c	ΔE_0 (eV)^d	R-factor (%)^e
Pristine Fe-N-C	Fe-N	3.9	1.92	8.9	1.7	0.01
Fe-N-C/SO_2	Fe-N	3.9	2.02	6.1	1.3	0.07
Fe-N-C/SeO_2	Fe-N	4.0	2.11	7.2	-3.7	0.10
Fe-N-C/TeO_2	Fe-N	4.1	2.06	6.3	3.5	0.08

[a]*N*, coordination number; [b]*R*, distance between absorber and backscatter atoms; [c]σ^2, Debye-Waller factor to account for both thermal and structural disorders; [d]ΔE_0, inner potential correction; [e]*R*-factor indicates the goodness of the fit. Error bounds (accuracies) that characterize the structural parameters obtained by EXAFS spectroscopy were estimated as $N \pm 20\%$; $R \pm 1\%$; $\sigma^2 \pm 20\%$; $\Delta E_0 \pm 20\%$. S_0^2 was fixed to 0.8. Fitting range: $2.5 \leq k$ (Å^{-1}) ≤ 11 and $1 \leq R$ (Å) ≤ 3.

Table S6. Fitting parameters of Fe *K*-edge EXAFS curves of pristine Fe-N-C and Fe-N-C/XO_2 with O_2 adsorption.

Sample	Path	N^a	R (Å)^b	σ^2 (× 10^{-3} Å^2)^c	ΔE_0 (eV)^d	R-factor (%)^e
Pristine Fe-N-C	Fe-N	3.9	1.93	4.3	6.9	0.08
	Fe-O	1.0	1.96	9.2		
Fe-N-C/SO_2	Fe-N	3.9	2.02	2.7	4.0	0.09
	Fe-O	1.1	1.94	3.6		
Fe-N-C/SeO_2	Fe-N	4.0	2.12	8.3	2.9	0.08
	Fe-O	1.0	1.90	2.9		
Fe-N-C/TeO_2	Fe-N	4.1	2.07	4.6	2.2	0.02
	Fe-O	1.0	1.95	4.3		

$^a N$, coordination number; $^b R$, distance between absorber and backscatter atoms; $^c\sigma^2$, Debye-Waller factor to account for both thermal and structural disorders; $^d\Delta E_0$, inner potential correction; $^e R$-factor indicates the goodness of the fit. Error bounds (accuracies) that characterize the structural parameters obtained by EXAFS spectroscopy were estimated as $N \pm 20\%$; $R \pm 1\%$; $\sigma^2 \pm 20\%$; $\Delta E_0 \pm 20\%$. S_0^2 was fixed to 0.8. Fitting range: $2.5 \leq k$ (Å^{-1}) ≤ 11 and $1 \leq R$ (Å) ≤ 3.

Table S7. Summary of Fe contents and number of FeN_4 active sites in pristine Fe-N-C and Fe-N-C/XO_2.

Sample	[a]Fe (wt%)	[b]Q_{strip} (C g^{-1})	[b]Site density (μmol g^{-1})
Pristine Fe-N-C	1.43	28.4	58.86
Fe-N-C/SO_2	1.41	31.6	65.49
Fe-N-C/SeO_2	1.43	28.1	58.54
Fe-N-C/TeO_2	1.45	29.9	61.96

[a] Determined by ICP-MS. [b] Determined by the nitrite stripping experiment.

Table S8. Comparison of ORR activity of Fe-N-C/SeO_2 with recently reported high performance catalysts in acid electrolyte.

Catalyst	Loading (mg cm^{-2})	electrolyte	$E_{1/2}$ (V)	J_k at 0.8V_{RHE} (mA cm^{-2})	Ref.
**Fe-N-C/SeO_2**	**0.8**	**0.5 M H_2SO_4**	**0.86**	**22.7**	**This work**
CNT/PC[a]	0.8	0.1 M $HClO_4$	0.79	4.5	[16]
SA-Fe-N nanosheets[b]	0.6	0.5 M H_2SO_4	0.81	-	[17]
Zn(elm)$_2$TPIP[c]	0.4	0.1 M $HClO_4$	0.78	2.5	[18]
Fe/SNC[d]	0.6	0.5 M H_2SO_4	0.77	-	[19]
Fe/N/C-SCN[e]	0.6	0.1 M $HClO_4$	0.83	13.8	[20]
CAPANI-Fe-NaCl[f]	0.6	0.1 M $HClO_4$	0.73	0.7	[21]
FeCo/C-800[g]	0.2	0.1 M $HClO_4$	0.76	1.4	[22]
(Fe, Fe)$_2$+N_2/H_2[h]	0.13	0.5 M H_2SO_4	0.75	0.41	[23]
Co-N-GA[i]	0.6	0.5 M H_2SO_4	0.73	-	[24]
Fe-NMCSs[j]	0.255	0.1 M $HClO_4$	0.74	-	[25]
Fe-N-C	0.8	0.1 M $HClO_4$	0.74	-	[26]

[a]CNT/PC: nanocomposite structure of carbon nanotube coated with thin layer of porphyrinic carbon. [b]SA-Fe-N nanosheets: the highly porous and sheet-like structure with atomically dispersed Fe-based active sites. [c]Zn(elm)$_2$TPIP: amorphous carbon material with a uniformly dispersed iron complex. [d]Fe/SNC: sulfur-doped Fe/N/C catalyst. [e]Fe/N/C-SCN: Fe/N/C catalyst with S-dopping and high surface area. [f]CAPANI-Fe-NaCl: N-doped carbon material with a 3D network structure. [g]FeCo/C-800: Fe and Co co-embedded porous carbon composite. [h](Fe, Fe)$_2$+N_2/H_2: Fe-N-C catalysts based on pyrolysis of porphyrins (FeTMPPCl) together with N_2/H_2. [i]Co-N-GA: a hierarchically porous Co–N functionalized graphene aerogel. [j]Fe-NMCSs: Fe-N-doped mesoporous carbon microspheres.

Supplementary References

[1] Y. Mun, S. Lee, K. Kim, S. Kim, S. Lee, J. W. Han, J. Lee, *J. Am. Chem. Soc.* **2019**, 141, 6254.

[2] M. Xiao, Y. Chen, J. Zhu, H. Zhang, X. Zhao, L. Gao, X. Wang, J. Zhao, J. Ge, Z. Jiang, S. Chen, C. Liu, W. Xing, *J. Am. Chem. Soc.* **2019**, 141, 17763.

[3] X. Wan, L. Xiaofang, L. Yongcheng, Y. Ronghai, Z. Lirong, Y. Wensheng, W. Hui, X. Ming, S. Jianglan, *Nat. Catal.* **2019**, 2, 259.

[4] L. Jiao, J. Li, L. L. Richard, Q. Sun, T. Stracensky, E. Liu, M. T. Sougrati, Z. Zhao, F. Yang, S. Zhong, H. Xu, S. Mukerjee, Y. Huang, D. A. Cullen, J. H. Park, M. Ferrandon, D. J. Myers, F. Jaouen, Q. Jia, *Nat. Mater.* **2021**, 20, 1385.

[5] N. Ramaswamy, U. Tylus, Q. Jia, S. Mukerjee, *J. Am. Chem. Soc.* **2013**, 135, 15443.

[6] S. Kattel, P. Atanassov, B. Kiefer, *J. Phys. Chem. C* **2012**, 116, 8161.

[7] J. Zhang, Z. Wang, Z. Zhu, Q. Wang, *J. Electrochem. Soc.* **2015**, 162, F796.

[8] T. Mineva, I. Matanovic, P. Atanassov, M.-T. Sougrati, L. Stievano, M. Clémancey, A. Kochem, J.-M. Latour, F. Jaouen, *ACS. Catal.* **2019**, 9, 9359.

[9] J. Li, M. T. Sougrati, A. Zitolo, J. M. Ablett, I. C. Oğuz, T. Mineva, I. Matanovic, P. Atanassov, Y. Huang, I. Zenyuk, A. Di Cicco, K. Kumar, L. Dubau, F. Maillard, G. Dražić, F. Jaouen, *Nat. Catal.* **2021**, 4, 10.

[10] U. I. Kramm, M. Lefevre, N. Larouche, D. Schmeisser, J. P. Dodelet, *J. Am. Chem. Soc.* **2014**, 136, 978.

[11] N. A. Fadzlillah, Y. B. Che Man, A. Rohman, *Int. J. Food. Prop.* **2014**, 17, 1275.

[12] N. Dukstiene, L. Tatariskinaite, M. Andrulevicius, *Mater. Sci-Poland.* **2010**, 28.

[13] X. Li, C.-S. Cao, S.-F. Hung, Y.-R. Lu, W. Cai, A. I. Rykov, S. Miao, S. Xi, H. Yang, Z. Hu, J. Wang, J. Zhao, E. E. Alp, W. Xu, T.-S. Chan, H. Chen, Q. Xiong, H. Xiao, Y. Huang, J. Li, T. Zhang, B. Liu, *Chem* **2020**, 6, 3440.

[14] J. Gu, C.-S. Hsu, L. Bai, H. M. Chen, X. Hu, *Science* **2019**, 364, 1091.

[15] V. A. Saveleva, K. Ebner, L. Ni, G. Smolentsev, D. Klose, A. Zitolo, E. Marelli, J. Li, M. Medarde, O. V. Safonova, M. Nachtegaal, F. Jaouen, U. I. Kramm, T. J. Schmidt, J. Herranz, *Angew. Chem. Int. Ed.* **2021**, 60, 11707.

[16] Y. J. Sa, D. J. Seo, J. Woo, J. T. Lim, J. Y. Cheon, S. Y. Yang, J. M. Lee, D. Kang, T. J. Shin, H. S. Shin, H. Y. Jeong, C. S. Kim, M. G. Kim, T. Y. Kim, S. H. Joo, *J. Am. Chem. Soc.* **2016**, 138, 15046.

[17] Z. Miao, X. Wang, M. C. Tsai, Q. Jin, J. Liang, F. Ma, T. Wang, S. Zheng, B. J. Hwang, Y. Huang, S. Guo, Q. Li, *Adv. Energy Mater.* **2018**, 8.

[18] D. Zhao, J. L. Shui, L. R. Grabstanowicz, C. Chen, S. M. Commet, T. Xu, J. Lu, D. J. Liu, *Adv. Mater.* **2014**, 26, 1093.

[19] H. Shen, E. Gracia-Espino, J. Ma, K. Zang, J. Luo, L. Wang, S. Gao, X. Mamat, G. Hu, T. Wagberg, S. Guo, *Angew. Chem. Int. Ed.* **2017**, 56, 13800.

[20] Y. C. Wang, Y. J. Lai, L. Song, Z. Y. Zhou, J. G. Liu, Q. Wang, X. D. Yang, C. Chen, W. Shi, Y. P. Zheng, M. Rauf, S. G. Sun, *Angew. Chem. Int. Ed.* **2015**, 54, 9907.

[21] W. Ding, L. Li, K. Xiong, Y. Wang, W. Li, Y. Nie, S. Chen, X. Qi, Z. Wei, *J. Am. Chem. Soc.* **2015**, 137, 5414.

[22] Q. Lin, X. Bu, A. Kong, C. Mao, F. Bu, P. Feng, *Adv. Mater.* **2015**, 27, 3431.

[23] U. I. Kramm, I. Herrmann-Geppert, J. Behrends, K. Lips, S. Fiechter, P. Bogdanoff, *J. Am. Chem. Soc.* **2016**, 138, 635.

[24] X. Fu, J. Y. Choi, P. Zamani, G. Jiang, M. A. Hoque, F. M. Hassan, Z. Chen, *ACS Appl. Mater. Interfaces* **2016**, 8, 6488.
[25] F. L. Meng, Z. L. Wang, H. X. Zhong, J. Wang, J. M. Yan, X. B. Zhang, *Adv. Mater.* **2016**, 28, 7948.
[26] S. Wang, M. Qiao, Y. Wang, Q. Wang, G. Hu, X. Mamat, s. zhang, *Angew. Chem. Int. Ed.* **2019**, 59, 2688.
